# Supplementary material for: Pathogenic POGZ mutation causes impaired cortical development and reversible autism-like phenotypes
Source: Nat Commun. 2020 Feb 26;11:859. doi: 10.1038/s41467-020-14697-z (PMC7044294; doi:10.1038/s41467-020-14697-z)
Supplement: Supplementary file 1 — Supplementary Information [file 41467_2020_14697_MOESM1_ESM.pdf]

Supplementary Information:

Pathogenic POGZ Mutation Causes Impaired Cortical Development and Reversible Autism-Like Phenotypes

Matsumura K. et al.

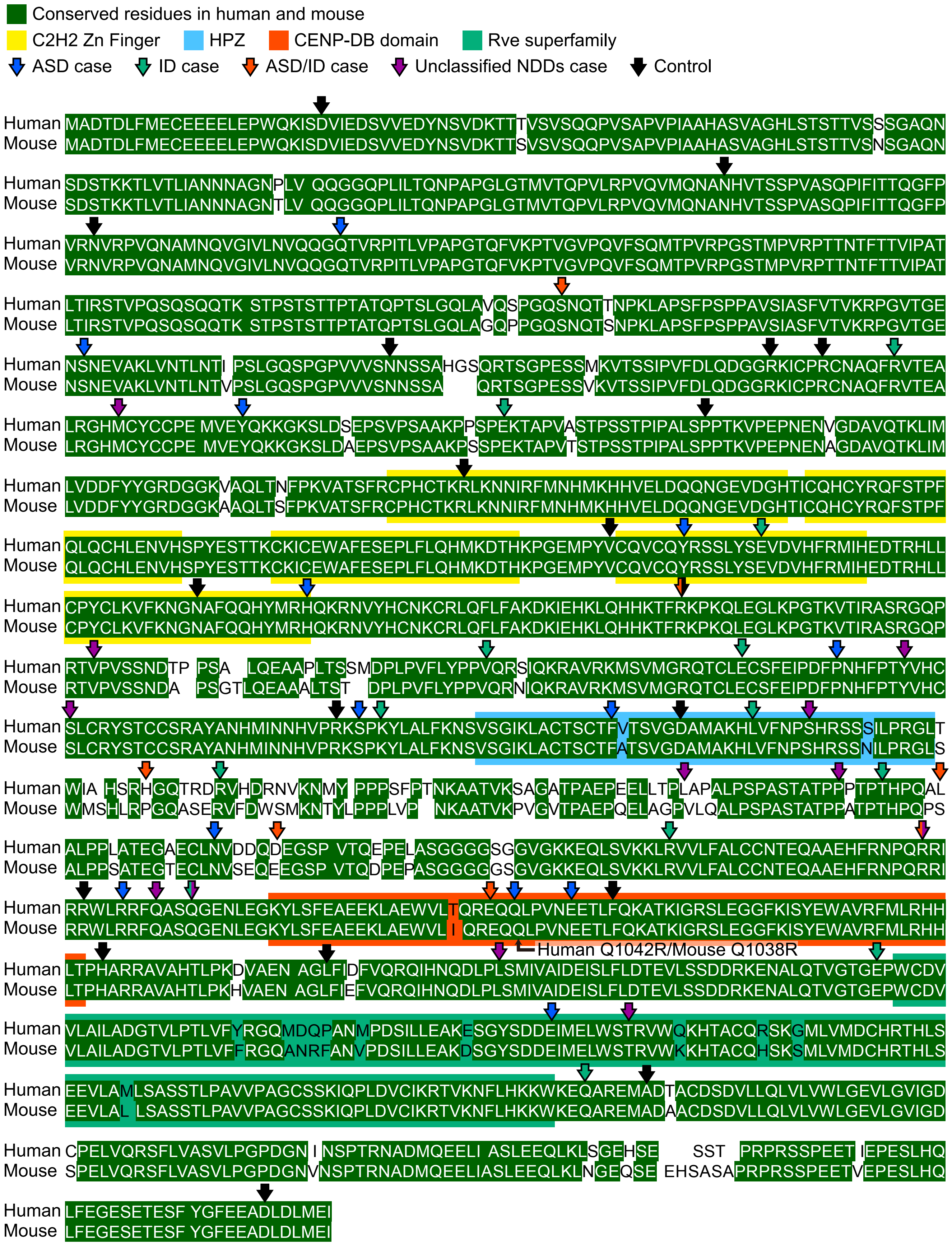

Supplementary Fig. 1 | **Amino acids sequence alignment of human and mouse POGZ.** Conserved residues between human POGZ (NP_055915.2) and mouse POGZ (NP_766271.2) are highlighted in green. Yellow boxes indicate C2H2 Zn Finger domains. The blue box indicates HPZ domain. The red box indicates CENP-DB domain. The green box indicates Rve-superfamily domain. Blue arrows indicate residues mutated in sporadic ASD cases. Green arrows indicate residues mutated in sporadic ID cases. Red arrows indicate residues mutated in sporadic ASD/ID cases. Purple arrows indicate residues mutated in sporadic unclassified NDDs cases. Black arrows indicate unaffected controls. Note that the mouse Q1038R mutation is corresponding to the human Q1042R mutation (indicated by an arrow in the CENP-DB domain).

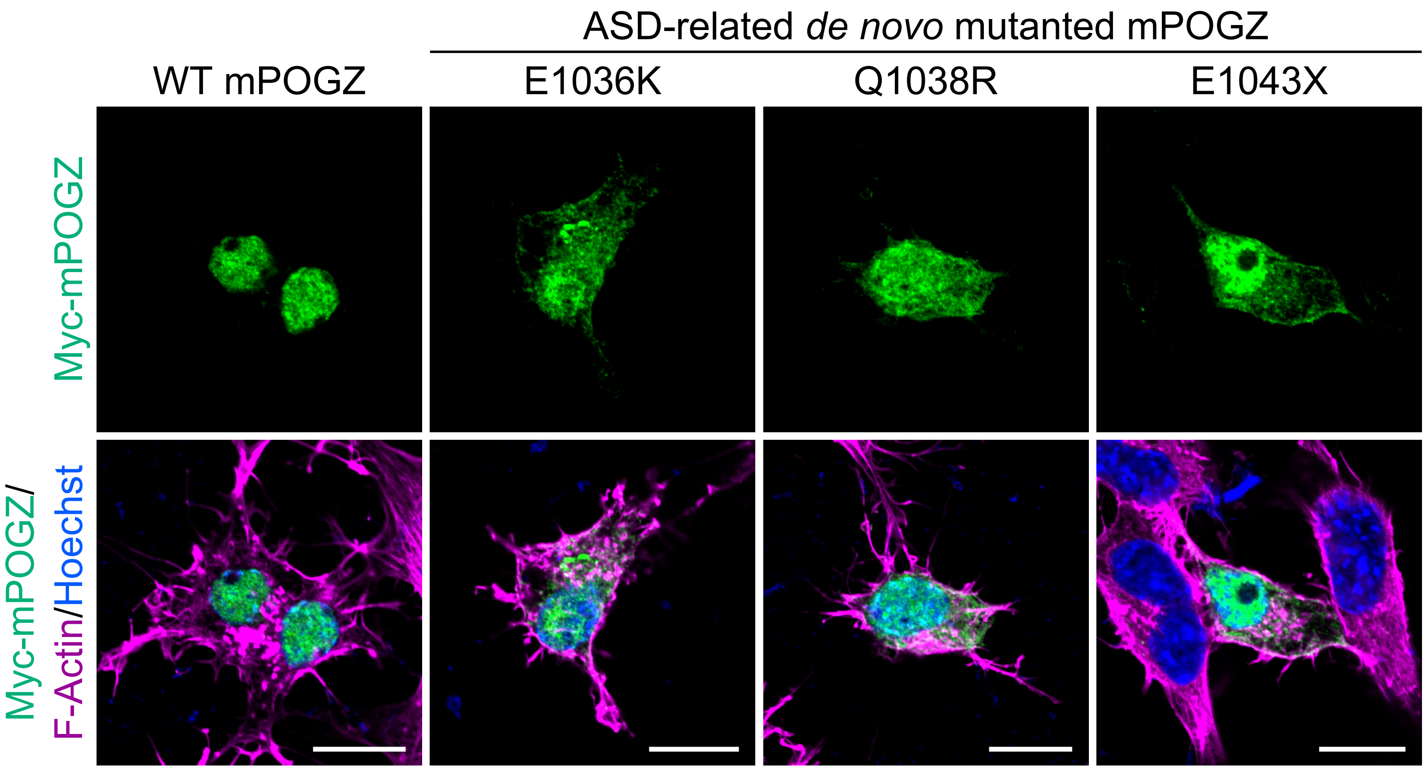

Supplementary Fig. 2 | **ASD-related *de novo* mutations in POGZ disrupt the cellular localization of the POGZ protein.** Myc-immunostaining and F-actin staining by Alexa Fluor 546-phalloidin showing nuclear localization of Myc-tagged overexpressed mouse (m) WT POGZ and disrupted cellular localization of Myc-tagged ASD-related *de novo* mutated mPOGZ variants in Neuro2a cells. WT, wild-type. Scale bars, 10 µm.

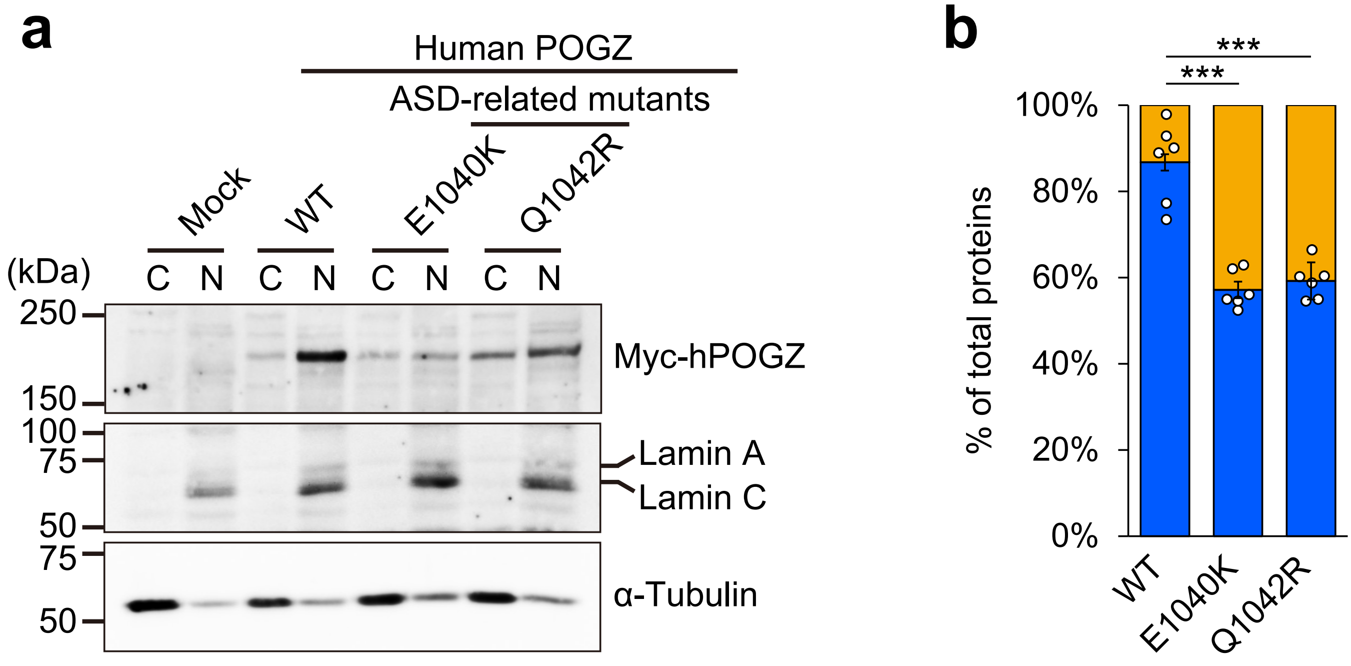

Supplementary Fig. 3 | **ASD-related *de novo* missense mutations in human POGZ impair the nuclear localization of the human POGZ protein. a**, ASD-related missense mutations disrupted the nuclear localization of Myc-tagged overexpressed human (h) POGZ in SHSY-5Y cells. C, cytosolic fraction; N, nuclear fraction; WT, wild-type. **b**, Quantification of Myc-hPOGZ in cytosolic and nuclear fractions (each n = 6). Note that the amino acid numbers are based on the human protein (NP_055915.2). One-way ANOVA with Bonferroni-Dunn *post hoc* tests; $F_{2,15} = 38.98$. ***$P < 0.001$. Data are presented as the mean ± s.e.m. The source data underlying Fig **a** are provided as a Source Data file.

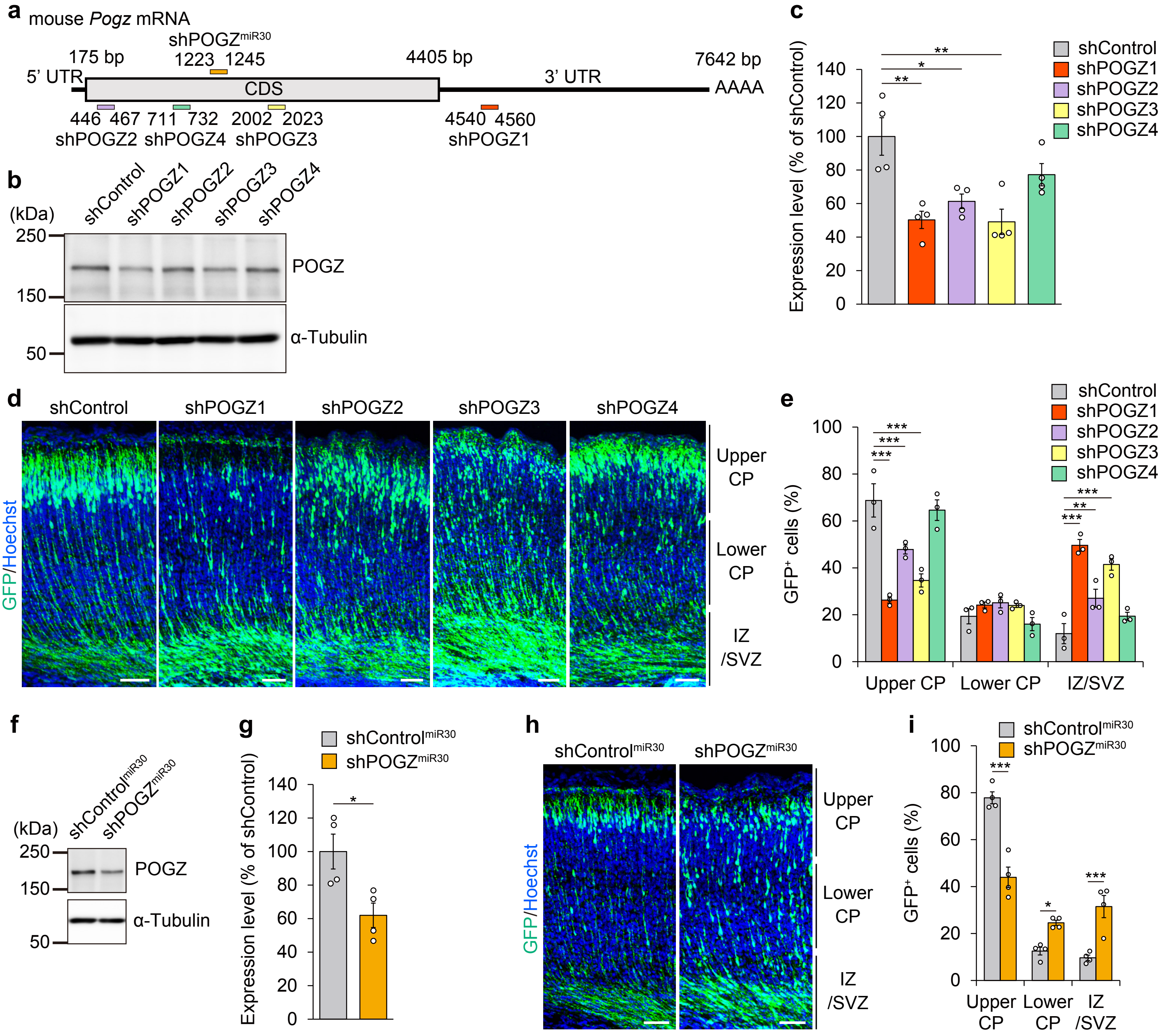

Supplementary Fig. 4 | **ShRNA- and shRNA^{miR30}- mediated knockdown of *Pogz*. a**, Schematic diagram of shRNAs and a miR30-based shRNA against mouse *Pogz* (NM_172683.3). **b**, Representative western blotting for endogenous POGZ in Neuro2a cells transfected with the plasmids expressing the indicated shRNA constructs against *Pogz*. **c**, Quantification of the expression levels of endogenous POGZ in Neuro2a cells transfected with the plasmids expressing indicated shRNA constructs against *Pogz* (each n = 4). **d**, Migration defects caused by shRNA-mediated knockdown of *Pogz* in E18.5 mouse cortices electroporated at E14.5. Scale bars, 50 µm. **e**, Quantification of GFP$^+$ cells in each layer (CP, cortical plate; IZ, intermediate zone; SVZ, subventricular zone; each n = 3). **f**, Representative western blotting for endogenous POGZ in Neuro2a cells transfected with the plasmid expressing the miR30-based shRNA construct against *Pogz*. **g**, Quantification of expression levels of endogenous POGZ in Neuro2a cells transfected with the plasmid expressing the miR30-based shRNA construct against *Pogz* (each n = 4). **h**, Migration defects caused by miR30-based shRNA-mediated knockdown of *Pogz* in E18.5 mouse cortices electroporated at E14.5. Scale bars, 50 µm. **i**, Quantification of GFP$^+$ cells in each layer (CP, cortical plate; IZ, intermediate zone; SVZ, subventricular zone; each n = 4). **c**, One-way ANOVA with Bonferroni-Dunn *post hoc* tests; **c**, $F_{4, 15} = 8.371$. **e**, **i**, Two-way repeated-measures ANOVA with Bonferroni-Dunn tests; **e**, $F_{8, 30} = 28.52$; i, $F_{2, 18} = 49.72$. **g**, Student's *t*-test. *$P < 0.05$, **$P < 0.01$, ***$P < 0.001$. Data are presented as the mean ± s.e.m. The source data underlying Figs **b** and **f** are provided as a Source Data file.

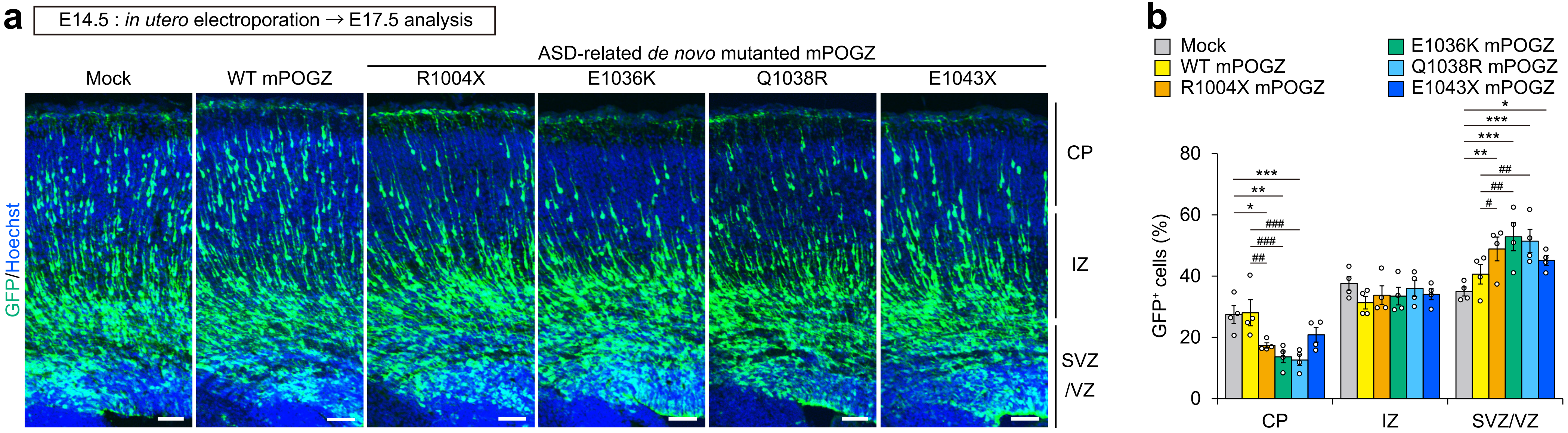

Supplementary Fig. 5 | **ASD-related POGZ mutations exhibit dominant negative effects on the function of endogenous POGZ. a**, Forced expression of the ASD-related mPOGZ mutants impaired the neuronal migration in E17.5 mouse cortices electroporated at E14.5. WT, wild-type; CP, cortical plate; IZ, intermediate zone; SVZ, subventricular zone; VZ, ventricular zone; E, embryonic day. Scale bars, 50 µm. **b**, Quantification of GFP^+ cells in each layer (each n = 4). Two-way repeated-measures ANOVA with Bonferroni-Dunn *post hoc* tests; **b**, $F_{10, 54} = 6.099$. $^{*}P < 0.05$, $^{**}P < 0.01$, $^{***}P < 0.001$, $^{\#}P < 0.05$, $^{\#\#}P < 0.01$, $^{\#\#\#}P < 0.001$ (vs WT mPOGZ). Data are presented as the mean ± s.e.m.

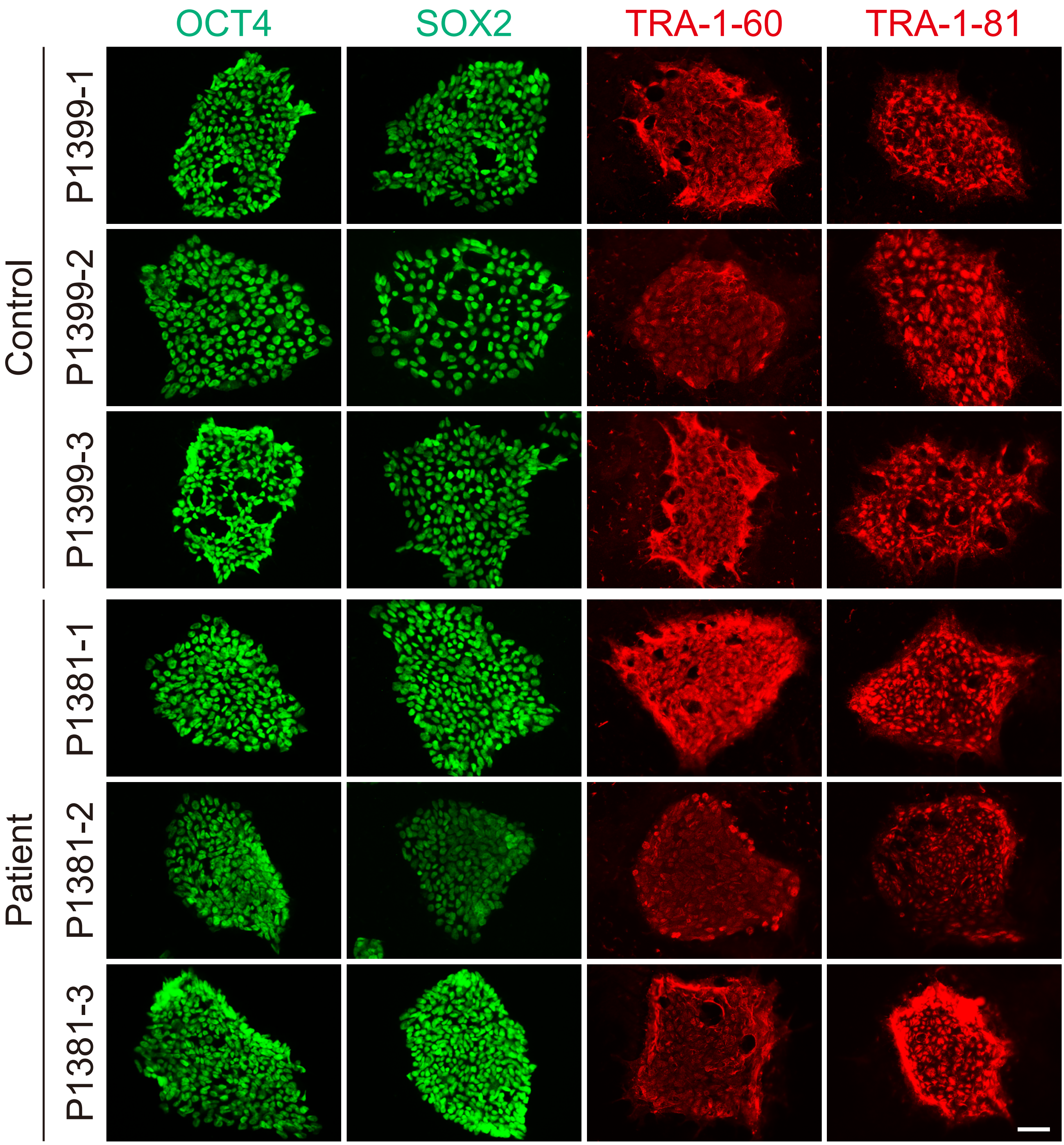

Supplementary Fig. 6 | **Generation of iPSC lines from a patient with ASD who carries the Q1042R mutation in POGZ and from an unaffected healthy control.** Representative images of iPSC lines. iPSC lines were stained for pluripotency markers (OCT-4A, SOX2, TRA-1-60 and TRA-1-81). Scale bar, 50 μm.

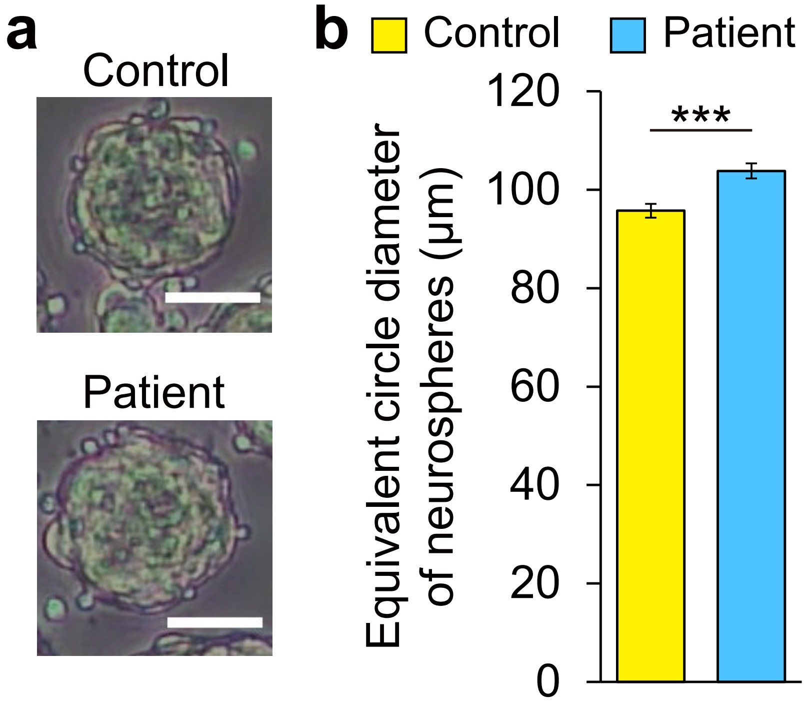

Supplementary Fig. 7 | **Increased size of patient-derived neurospheres. a**, Representative images of neurospheres derived from the patient and unaffected healthy control. Scale bars, 50 µm. **b**, Quantification of the size of neurospheres derived from the patient and unaffected healthy control (control, n = 681; patient, n = 537). **b**, Student's *t*-test. ***$P < 0.001$. Data are presented as the mean ± s.e.m. The source data underlying Fig **b** are provided as a Source Data file.

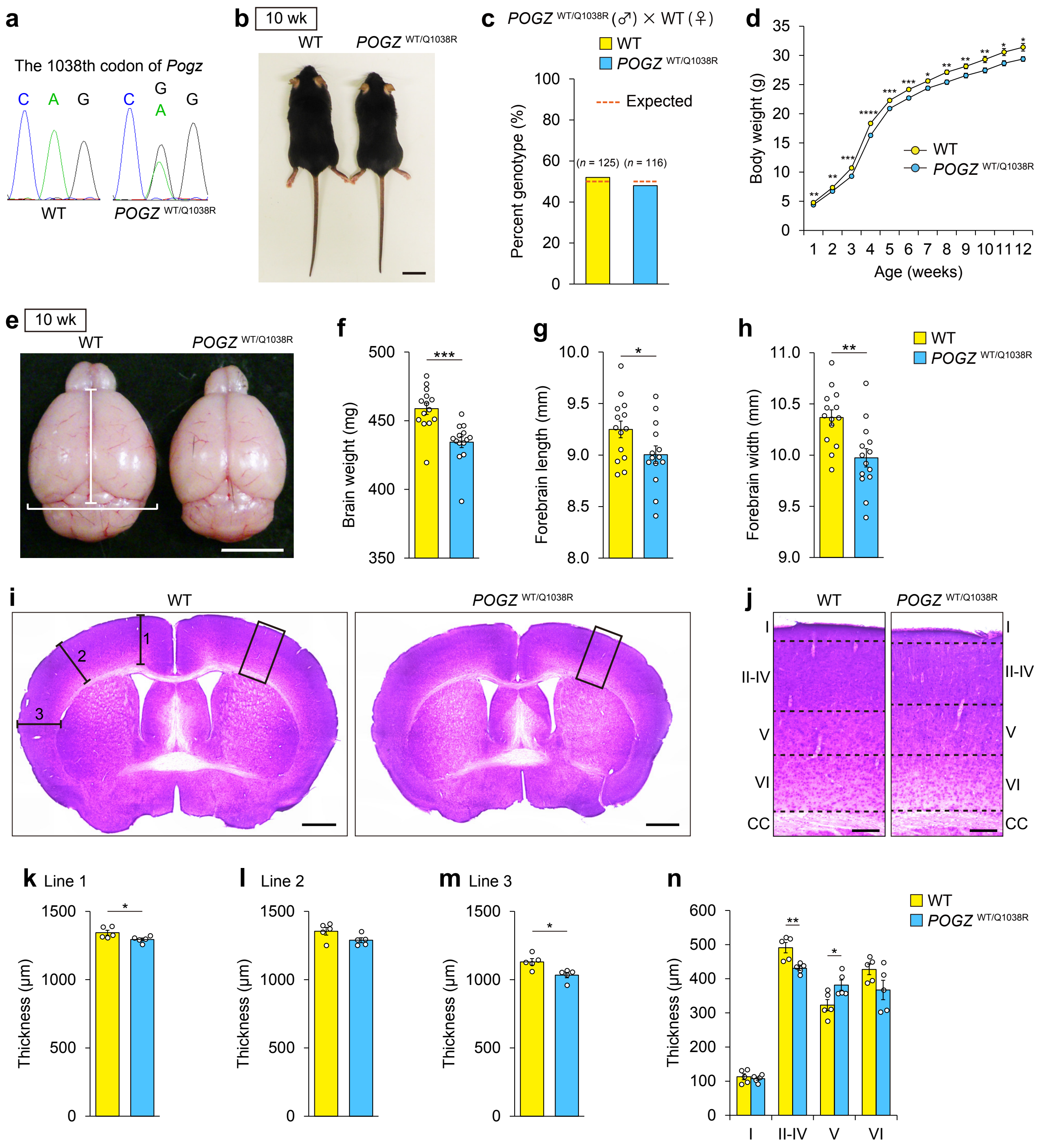

Supplementary Fig. 8 | **Generation of *POGZ*$^{\mathbf{WT/Q1038R}}$ mice. a**, The A is substituted with G in codon 1038 of the mouse *Pogz* gene. **b**, Decreased body size in *POGZ*$^{WT/Q1038R}$ mice at 10 wk. wk, week-old. Scale bar, 2 cm. **c**, Genotyping of 1 wk offspring from crosses between male *POGZ*$^{WT/Q1038R}$ mice and female WT mice yielded the expected Mendelian ratio of WT and *POGZ*$^{WT/Q1038R}$ mice (n = 30 pairs). **d**, Quantification of body weight in WT and *POGZ*$^{WT/Q1038R}$ mice (WT, n = 20; *POGZ*$^{WT/Q1038R}$, n = 21). **e**, Decreased brain size in *POGZ*$^{WT/Q1038R}$ mice at 10 wk. Scale bar, 5 mm. Forebrain length and width are indicated by white lines. **f**, Quantification of brain weight (each n = 14). **g**, Quantification of forebrain length (each n = 14). **h**, Quantification of forebrain width (each n = 14). **i**, Representative coronal sections of WT and *POGZ*$^{WT/Q1038R}$ brains at 10 wk visualized with hematoxylin and eosin (HE) staining at Bregma 0 mm. We measured the length of each line as thickness of the cortex. Scale bars, 1 mm. **j**, Magnifications of the areas outlined with black boxes in **i**. Scale bars, 200 µm. **k-m**, Quantification of thickness of the cortex at line 1 (**k**), line 2 (**l**) and line 3 (**m**) shown in **i** (each n = 5). **n**, Quantification of thickness of each cortical layer shown in **j** (each n = 5). WT, wild-type; wk, week-old. **d**, **n**, Two-way repeated-measures ANOVA with Bonferroni-Dunn *post hoc* tests; **d**, $F_{11, 468} = 1.089$; **n**, $F_{3, 32} = 6.717$. **f**, **g**, **h**, **k**, **l**, **m**, Student's *t*-test. *$P < 0.05$, **$P < 0.01$, ***$P < 0.001$. Data are presented as the mean ± s.e.m. The source data underlying Fig **d** are provided as a Source Data file.

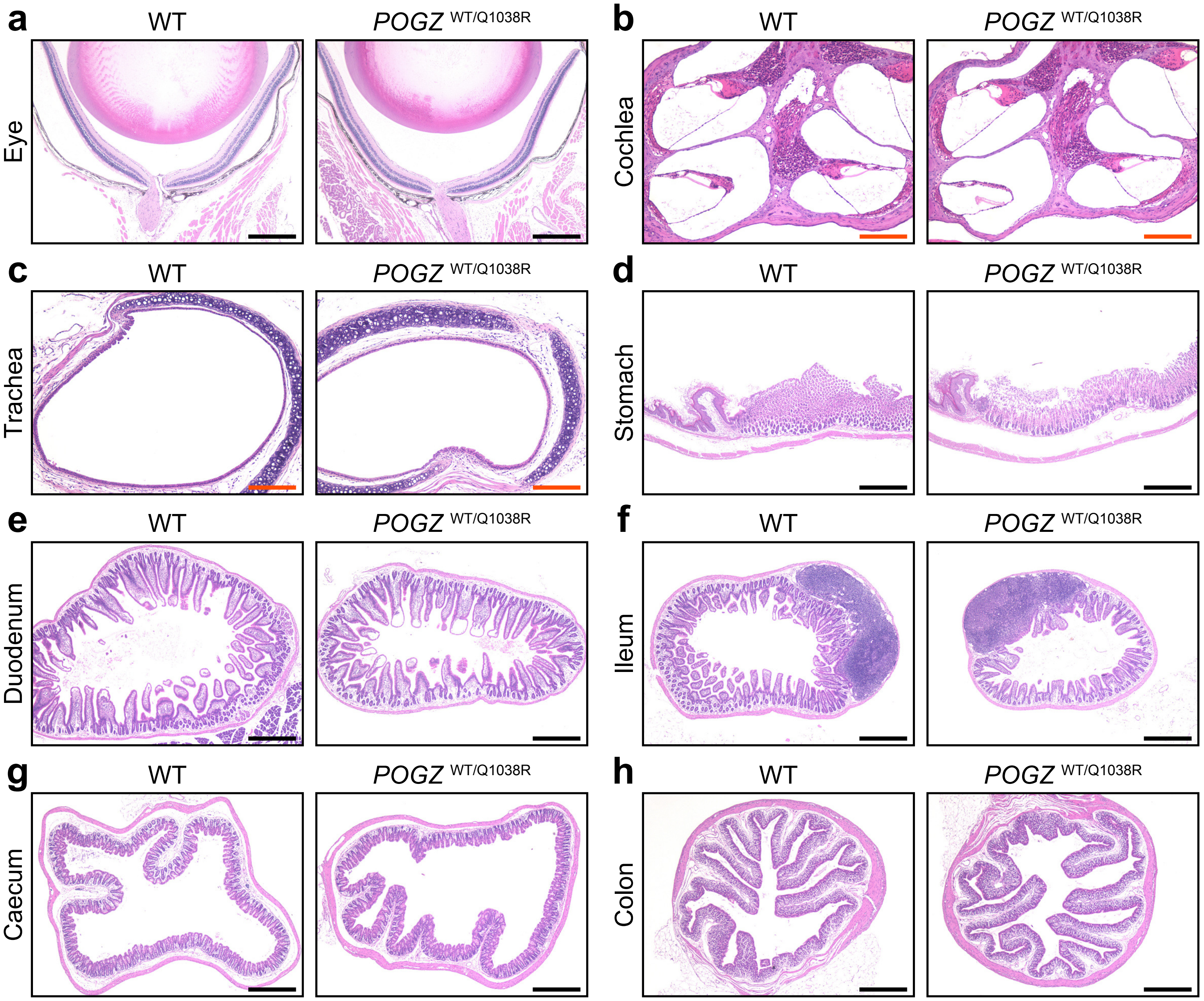

Supplementary Fig. 9 | **Hematoxylin and eosin (HE) staining of peripheral organs of *POGZ*$^{\mathrm{WT/Q1038R}}$ mice. a-j**, *POGZ*$^{\mathrm{WT/Q1038R}}$ mice did not exhibit any significant changes in peripheral organs, including eye (**a**), cochlea (**b**), trachea (**c**), stomach (**d**), duodenum (**e**), ileum (**f**), caecum (**g**) and colon (**h**), compared to WT mice. WT, wild-type. Black scale bars, 500 µm; red scale bars, 200 µm.

a

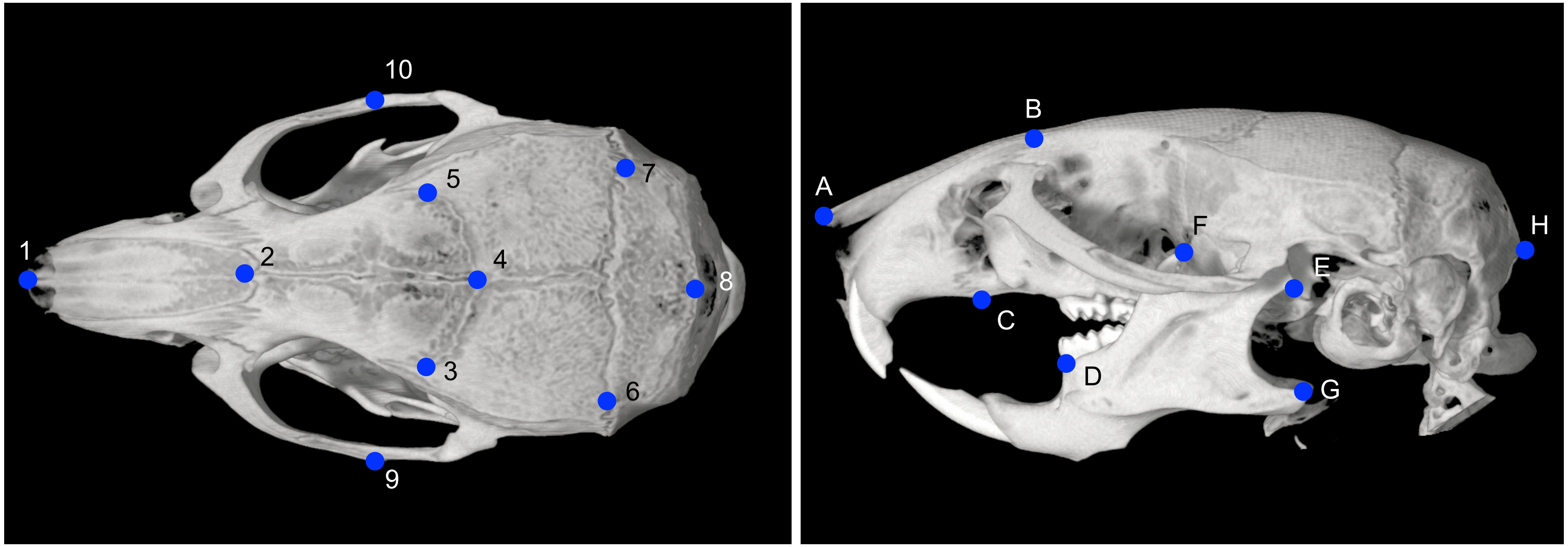

b

landmark	WT	*POGZ*$^{WT/Q1038R}$	*P* value
1 and 2	7.42 ± 0.07	7.12 ± 0.16	NS
1 and 4	15.42 ± 0.05	14.69 ± 0.30	NS
1 and 8	22.57 ± 0.08	21.45 ± 0.28	NS
3 and 5	6.11 ± 0.13	5.97 ± 0.09	NS
6 and 7	8.39 ± 0.13	7.86 ± 0.09	NS
9 and 10	12.28 ± 0.12	11.77 ± 0.21	NS
A and H	23.41 ± 0.26	23.27 ±0.32	NS
B and C	5.84 ± 0.13	5.73 ± 0.08	NS
D and E	8.25 ± 0.08	8.10 ± 0.12	NS
F and G	6.47 ± 0.12	6.56 ± 0.05	NS

c

	WT	*POGZ*$^{WT/Q1038R}$	*P* value
1 and 2 / 1 and 8	0.33	0.33	NS
1 and 4 /1 and 8	0.68	0.68	NS
3 and 5 / 1 and 8	0.27	0.28	NS
6 and 7 / 1 and 8	0.37	0.37	NS
9 and 10 / 1 and 8	0.54	0.55	NS
B and C / A and H	0.25	0.25	NS
F and G / D and E	0.78	0.81	NS

Supplementary Fig. 10 | **CT analysis of the skull of *POGZ*$^{WT/Q1038R}$ mice. a**, Schematics of landmarks in the mouse skull. The top view of the CT image (*left*); Temporal side view of the CT image (*right*). Numerals and letters indicate the anatomical landmarks in **b**. The schematics are created by us (Shibuya and Tamura). **b**, Linear distances (mm) between landmarks in WT and *POGZ*$^{WT/Q1038R}$ mice. Anatomical landmarks for the skull were referred to the previous report (Maga, A.M. *et al*., *Front. Physiol.* **6**, 92 (2015)) (WT, n = 6; *POGZ*$^{WT/Q1038R}$, n =5). **c**, Linear distance ratios between landmarks of WT and *POGZ*$^{WT/Q1038R}$ mice (WT, n = 6; *POGZ*$^{WT/Q1038R}$, n =5). WT, wild-type; NS, not significant. **b**, **c**, Welch's *t*-test. Data are presented as the mean ± s.e.m.

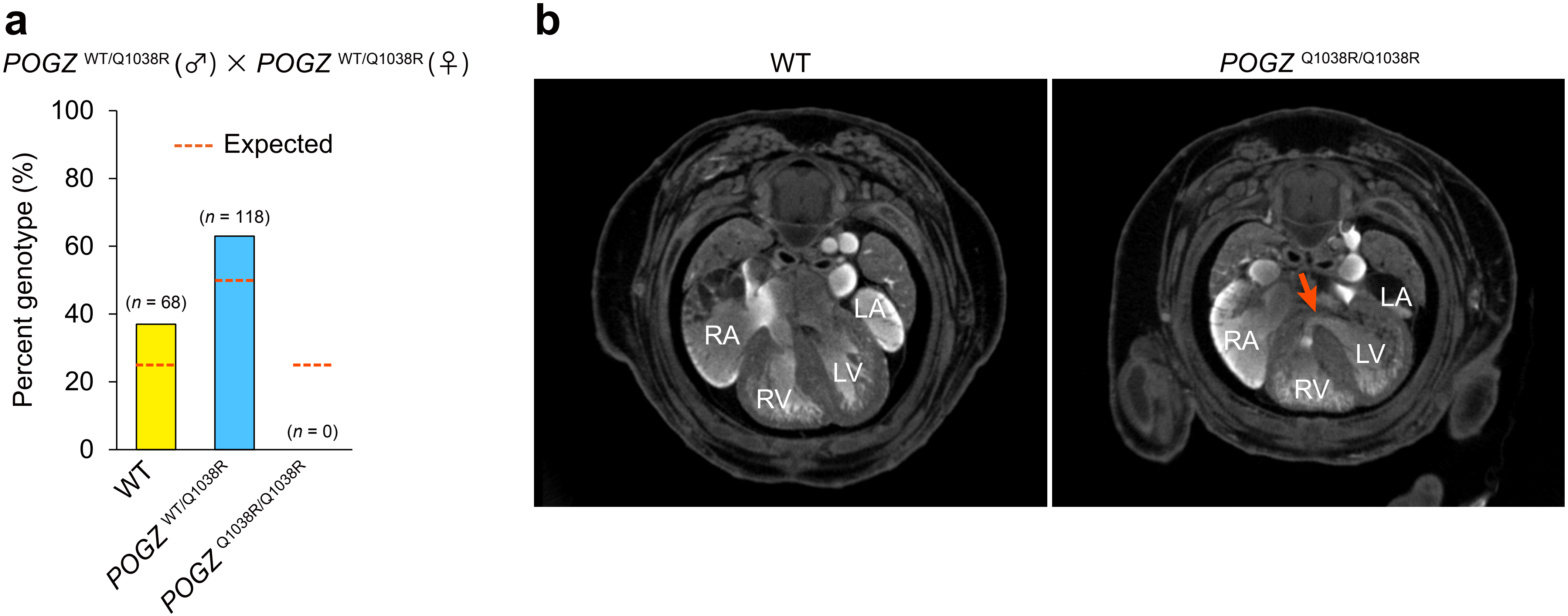

Supplementary Fig. 11 | **Embryonic lethality of homozygous *POGZ*$^{\mathbf{Q1038R/Q1038R}}$ mice. a**, Genotyping of 1-week-old offspring from intercrosses of *POGZ*$^{WT/Q1038R}$ mice yielded no homozygous *POGZ*$^{Q1038R/Q1038R}$ mice (n = 25 pairs). **b**, Representative images of the CT analysis of WT and *POGZ*$^{Q1038R/Q1038R}$ mice at E15.5 (each n = 4). The red arrow indicates ventricular septal defects. WT, wild-type; LA, Left atrium; LV, left ventricle; RA, right atrium; RV, right ventricle.

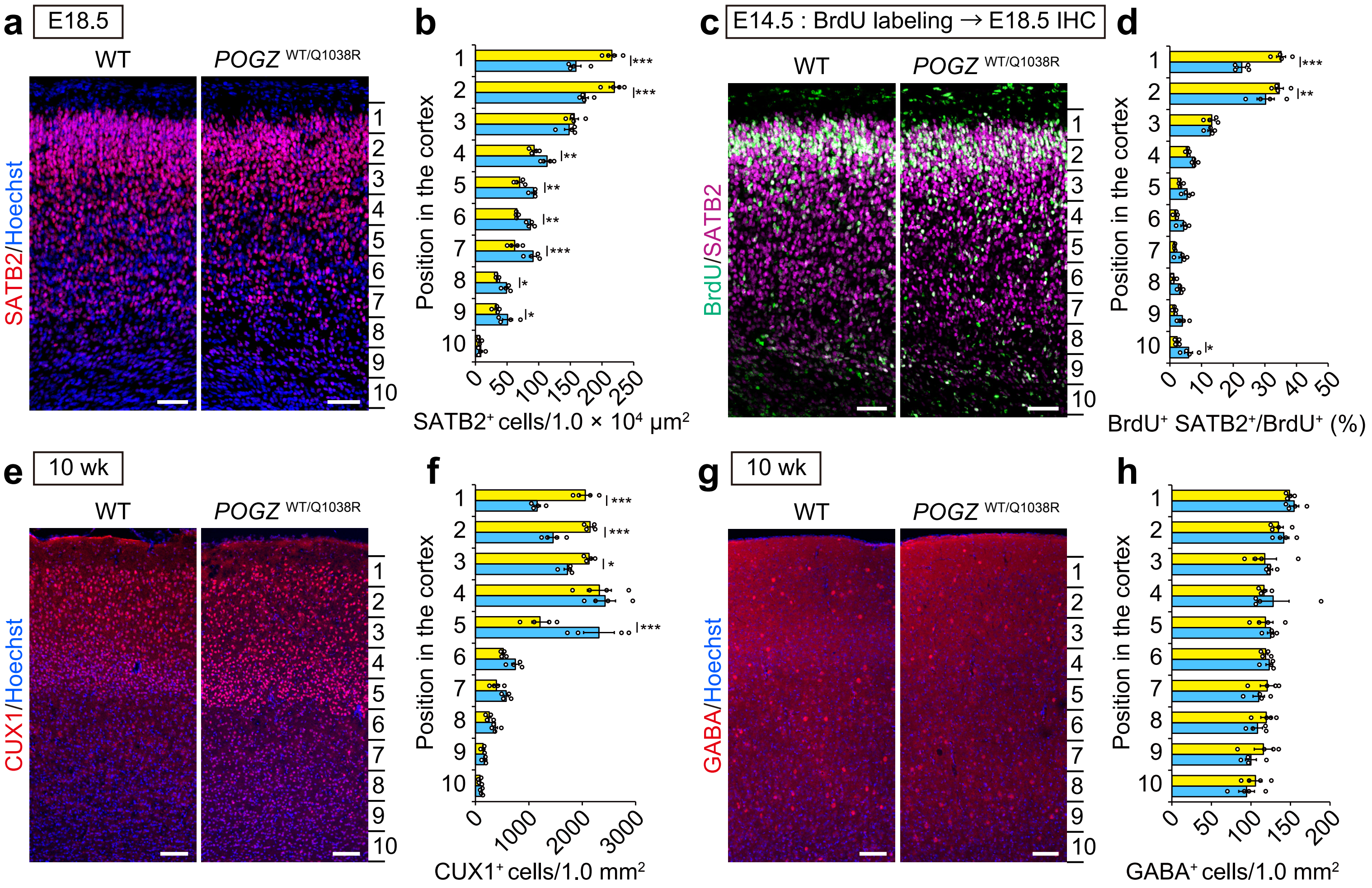

Supplementary Fig. 12 | **Impaired cortical neuronal development in *POGZ*$^{WT/Q1038R}$ mice. a**, SATB2 immunostaining showing the abnormal distribution of SATB2$^+$ cortical excitatory neurons in *POGZ*$^{WT/Q1038R}$ mice at E18.5. Scale bars, 50 µm. WT, wild-type; E, embryonic day. **b**, Distribution of SATB2$^+$ neurons in ten equal bins (CP 1 to IZ/SVZ 10) within the developing cortex (each n = 4). The cortex was divided into 10 layers to form the bins. **c**, BrdU and SATB2 immunostaining in *POGZ*$^{WT/Q1038R}$ mice at E18.5. Scale bars, 50 µm. IHC, immunohistochemistry. **d**, Distribution of SATB2$^+$ BrdU$^+$ neurons in ten equal bins (CP 1 to IZ/SVZ 10) within the developing cortex (each n = 4). The cortex was divided into 10 layers to form the bins. Scale bars, 50 µm. **e**, CUX1 immunostaining showing the abnormal distribution of CUX1$^+$ cortical excitatory neurons in the adult *POGZ*$^{WT/Q1038R}$ mice (10 weeks old). Scale bars, 100 µm. wk, week-old. **f**, Distribution of CUX1$^+$ neurons in ten equal bins (CP 1 to IZ/SVZ 10) of the adult cortex (each n = 4). The cortex was divided into 10 layers to form the bins. **g**, GABA immunostaining showing the normal distribution of GABA$^+$ inhibitory neurons in the adult *POGZ*$^{WT/Q1038R}$ mice (10 weeks old). Scale bars, 100 µm. **h**, Distribution of GABA$^+$ neurons in ten equal bins (CP 1 to IZ/SVZ 10) of the adult cortex (each n = 4). The cortex was divided into 10 layers to form the bins. CP, cortical plate; IZ, intermediate zone; SVZ, subventricular zone. **b**, **d**, **f**, **h**, Two-way repeated-measures ANOVA with Bonferroni-Dunn *post hoc* tests; **b**, $F_{9, 60} = 17.03$; **d**, $F_{9, 60} = 12.17$; **f**, $F_{9, 60} = 12.62$; **g**, $F_{9, 60} = 0.706$. *$P < 0.05$, **$P < 0.01$, ***$P < 0.001$. Data are presented as the mean ± s.e.m.

a

Enriched transcriptional networks in NSCs derived from the Patient and *POGZ*$^{WT/Q1038R}$ mice

ID	Name	Patient		*POGZ*$^{WT/Q1038R}$ mice	
		p value	FDR q value	p value	FDR q value
GO:0007399	nervous system development	8.82E-11	2.48E-08	1.32E-05	2.19E-04
GO:0051239	regulation of multicellular organismal process	4.93E-09	6.93E-07	9.06E-10	5.09E-08
GO:0048856	anatomical structure development	1.19E-08	9.43E-07	2.13E-07	6.66E-06
GO:0022008	neurogenesis	1.34E-08	9.43E-07	1.20E-03	8.88E-03
GO:0055085	transmembrane transport	1.99E-08	1.12E-06	2.48E-06	4.98E-05
GO:0048699	generation of neurons	6.25E-08	2.93E-06	1.69E-03	1.06E-02
GO:0007275	multicellular organism development	8.15E-08	2.97E-06	4.04E-08	1.42E-06
GO:0009653	anatomical structure morphogenesis	8.47E-08	2.97E-06	1.05E-03	7.93E-03
GO:0048731	system development	1.05E-07	3.28E-06	2.74E-08	1.10E-06
GO:0034220	ion transmembrane transport	1.98E-07	5.57E-06	3.49E-07	9.80E-06
GO:0032502	developmental process	2.69E-07	6.88E-06	1.48E-06	3.20E-05
GO:0007267	cell-cell signaling	4.64E-07	1.08E-05	2.40E-15	6.74E-13
GO:0030182	neuron differentiation	5.02E-07	1.08E-05	1.48E-03	1.03E-02
GO:0051240	positive regulation of multicellular organismal process	6.12E-07	1.23E-05	1.96E-08	9.17E-07
GO:0006811	ion transport	1.00E-06	1.88E-05	6.49E-10	4.56E-08
GO:0048869	cellular developmental process	3.04E-06	5.34E-05	3.01E-04	2.73E-03
GO:2000026	regulation of multicellular organismal development	5.10E-06	8.21E-05	2.19E-05	3.07E-04
GO:0007166	cell surface receptor signaling pathway	5.26E-06	8.21E-05	6.70E-04	5.71E-03
GO:0007155	cell adhesion	5.93E-06	8.76E-05	1.08E-11	1.01E-09
GO:0050793	regulation of developmental process	6.75E-06	9.48E-05	2.10E-04	2.03E-03
GO:0006812	cation transport	7.08E-06	9.48E-05	6.37E-07	1.63E-05
GO:0022610	biological adhesion	8.63E-06	1.10E-04	5.46E-12	7.66E-10
GO:0030154	cell differentiation	9.85E-06	1.20E-04	1.06E-04	1.14E-03
GO:0048646	anatomical structure formation involved in morphogenesis	1.24E-05	1.44E-04	1.24E-03	8.96E-03
GO:0048468	cell development	1.28E-05	1.44E-04	1.17E-04	1.22E-03
GO:0006928	movement of cell or subcellular component	3.49E-05	3.77E-04	3.04E-03	1.74E-02
GO:0048513	animal organ development	4.93E-05	4.77E-04	6.97E-04	5.76E-03
GO:0045595	regulation of cell differentiation	5.50E-05	5.15E-04	1.95E-03	1.17E-02
GO:0051094	positive regulation of developmental process	6.97E-05	6.32E-04	2.02E-05	2.99E-04
GO:0065008	regulation of biological quality	9.70E-05	8.26E-04	1.21E-06	2.83E-05
GO:0023051	regulation of signaling	1.66E-04	1.30E-03	1.53E-03	1.03E-02

b

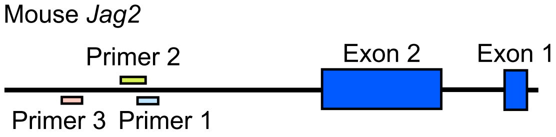

c

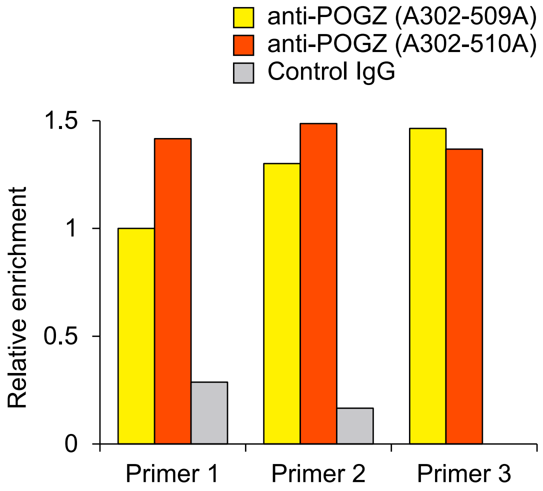

Supplementary Fig. 13 | **GO Annotation analysis and ChIP assay in NSCs. a.** Common gene ontology (GO) annotations (molecular function) for the differentially expressed genes between NSCs derived from the unaffected healthy control and the patient carrying the Q1042R mutation of POGZ, and E16.5 embryonic cortex of WT and *POGZ*$^{WT/Q1038R}$ mice by the ToppGene Suite (https://toppgene.cchmc.org/). **b.** Schematic diagram of qPCR amplicons of the primers for the mouse *Jag2* (NC_000078.6) promoter. **c.** ChIP-qPCR assay for the quantification of relative enrichment of the genomic region in the mouse *Jag2* promoter.

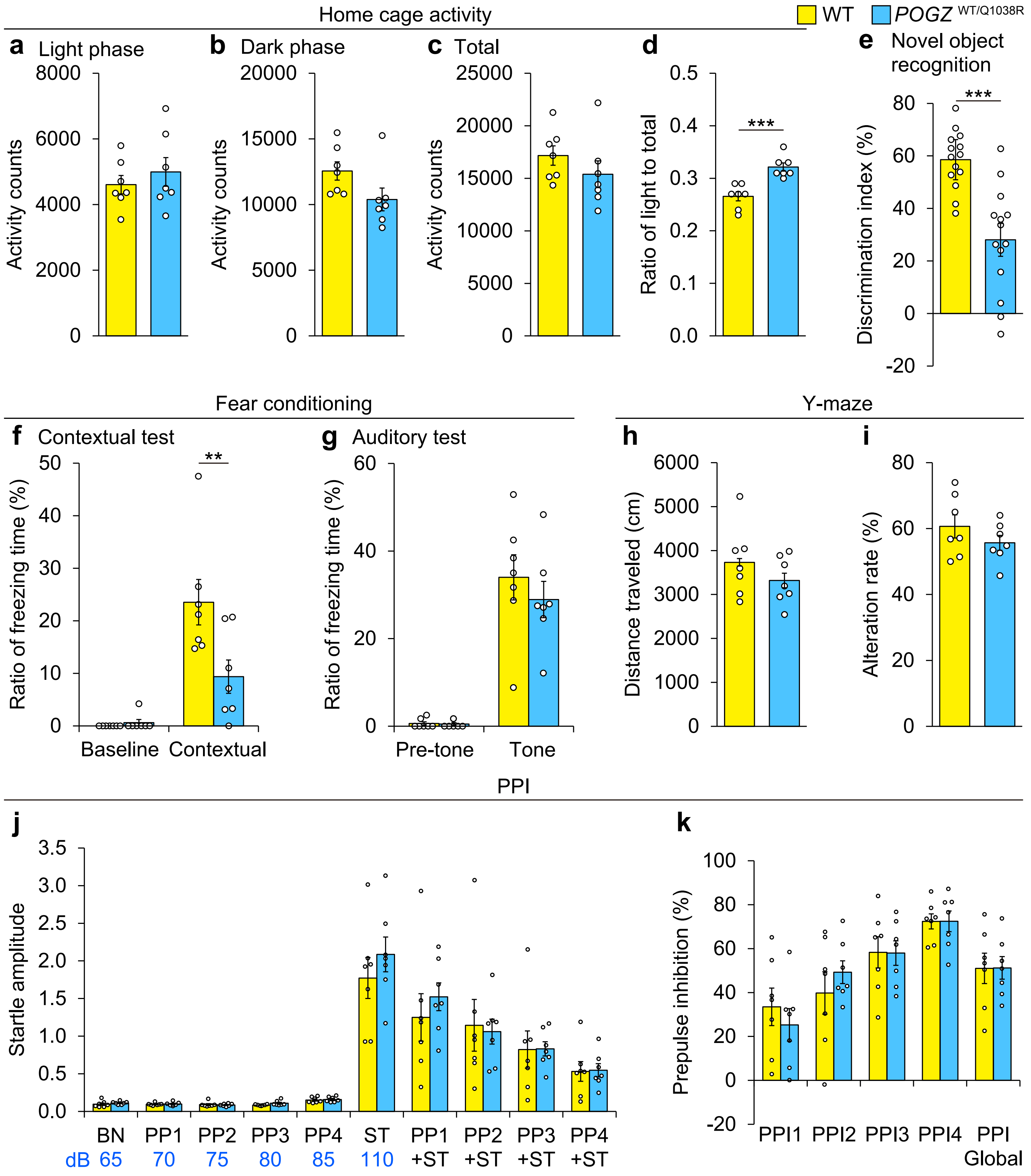

Supplementary Fig. 14 | **Behavioral abnormalities of heterozygous *POGZ*$^{WT/Q1038R}$ mice. a-d**, Home-cage activity of WT and *POGZ*$^{WT/Q1038R}$ mice (each n = 7). **e**, Impaired novel object recognition in *POGZ*$^{WT/Q1038R}$ mice (each n = 14). **f**, Impaired contextual fear memory in *POGZ*$^{WT/Q1038R}$ mice (each n = 7). **g**, Normal auditory fear memory in *POGZ*$^{WT/Q1038R}$ mice (each n = 7). **h**, **i**, Normal locomotion (**h**) and alteration ratio (**i**) in *POGZ*$^{WT/Q1038R}$ mice in the Y-maze test (each n = 7). **j**, **k**, Normal PPI in *POGZ*$^{WT/Q1038R}$ mice (each n = 7). WT, wild-type; PPI, prepulse inhibition; BN, background noise. **a-e**, **h**, **i**, One-way ANOVA with Bonferroni-Dunn *post hoc* tests; **a**, $F_{1,12} = 0.558$; **b**, $F_{1,12} = 3.821$; **c**, $F_{1,12} = 1.282$; **d**, $F_{1,12} = 23.05$; **e**, $F_{1,26} = 24.76$; **h**, $F_{1,12} = 1.272$; **i**, $F_{1,12} = 1.428$. **f**, **g**, **k**, Two-way ANOVA with Bonferroni-Dunn *post hoc* tests; **f**, $F_{1,24} = 7.482$; **g**, $F_{1,24} = 0.558$; **k**, $F_{4,60} = 0.448$. $^{**}P < 0.01$, $^{***}P < 0.001$. Data are presented as the mean ± s.e.m.

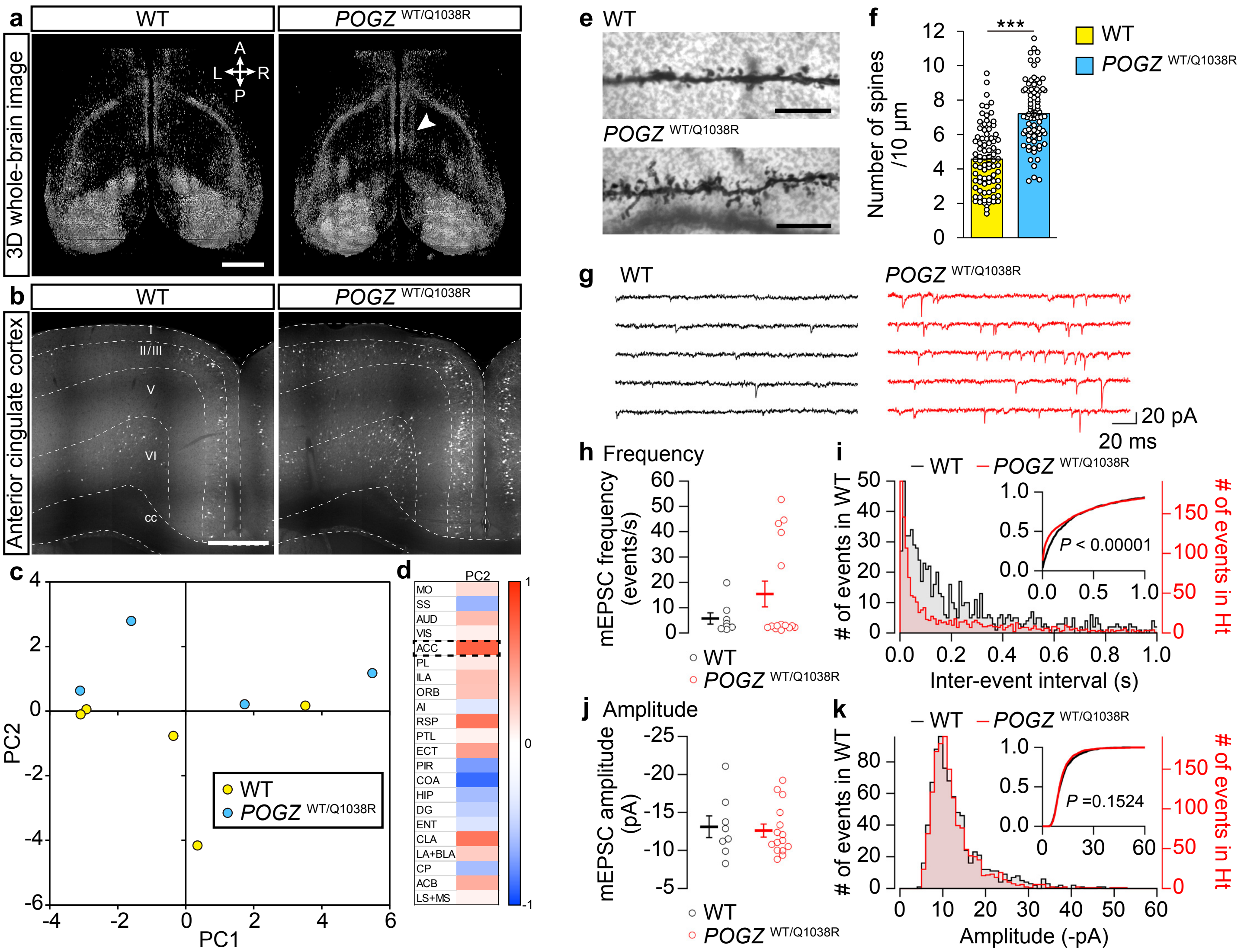

Supplementary Fig. 15 | **Increased cortical neuronal activity in *POGZ*$^{WT/Q1038R}$ mice.** **a**, Representative brain regions showing social-interaction-induced activation of Arc-dVenus fluorescence in adult WT and *POGZ*$^{WT/Q1038R}$ mice. Scale bar, 2 mm. **b**, Representative images of social-interaction-induced activation of Arc-dVenus fluorescence in the anterior cingulate cortex in adult WT and *POGZ*$^{WT/Q1038R}$ mice. Scale bar, 500 μm. **c**, Locations of individual WT and *POGZ*$^{WT/Q1038R}$ mice projected in principal component (PC) space defined by the first two PCs (in arbitrary PC units). **d**, Loadings for PC2 (in arbitrary PC weight units). Note the especially large contribution of the anterior cingulate cortex (ACC) to PC2 (dashed box). MO, motor cortex; SS, somatosensory cortex; AUD, auditory cortex; VIS, visual cortex; PL, prelimbic cortex; ILA, infralimbic cortex; ORB, orbitofrontal cortex; Al, agranular insular cortex; RSP, retrosplenial cortex; PTL, posterior parietal association cortex; ECT, ectorhinal cortex; PIR, piriform cortex; COA, cortical amygdala; HIP, hippocampus excluding the dentate gyrus; DG, dentate gyrus; ENT, entorhinal cortex; CLA, claustrum; LA+BLA, lateral amygdala + basolateral amygdala; CP, caudoputamen; ACB, nucleus accumbens; LS+MS, lateral septum + medial septum. **e**, Golgi-staining showing increased spine density in the ACC in *POGZ*$^{WT/Q1038R}$ mice. Scale bars, 10 μm. **f**, Quantification of the spine density in the ACC (WT, n = 81 dendrites; *POGZ*$^{WT/Q1038R}$, n = 80 dendrites). **g**, Representative traces of mEPSCs obtained from the ACC of adult WT and *POGZ*$^{WT/Q1038R}$ mice. **h**, **j**, Summary of mEPSC frequency (**h**) and amplitude (**j**) recorded in each neuron (WT, n = 8 neurons; *POGZ*$^{WT/Q1038R}$, n = 15 neurons). Note that averaged mEPSC frequency in *POGZ*$^{WT/Q1038R}$ mice tended to be higher than that in WT mice. **i**, **k**, The distribution of the inter-event interval (**i**, WT, n = 792 events; *POGZ*$^{WT/Q1038R}$, n = 1,485 events) and the amplitude (**k**, WT, n = 800 events; *POGZ*$^{WT/Q1038R}$, n = 1,500 events) of mEPSCs. The cumulative probability plots described in the inset show a significant shift of the distribution of inter-event interval toward shorter intervals in *POGZ*$^{WT/Q1038R}$ mice. **f**, Student's *t*-test. **h**, **j**, Mann-Whitney *U* test. **i**, **k**, Kolmogorov-Smirnov test. ***$P < 0.001$. Data are presented as the mean ± s.e.m.

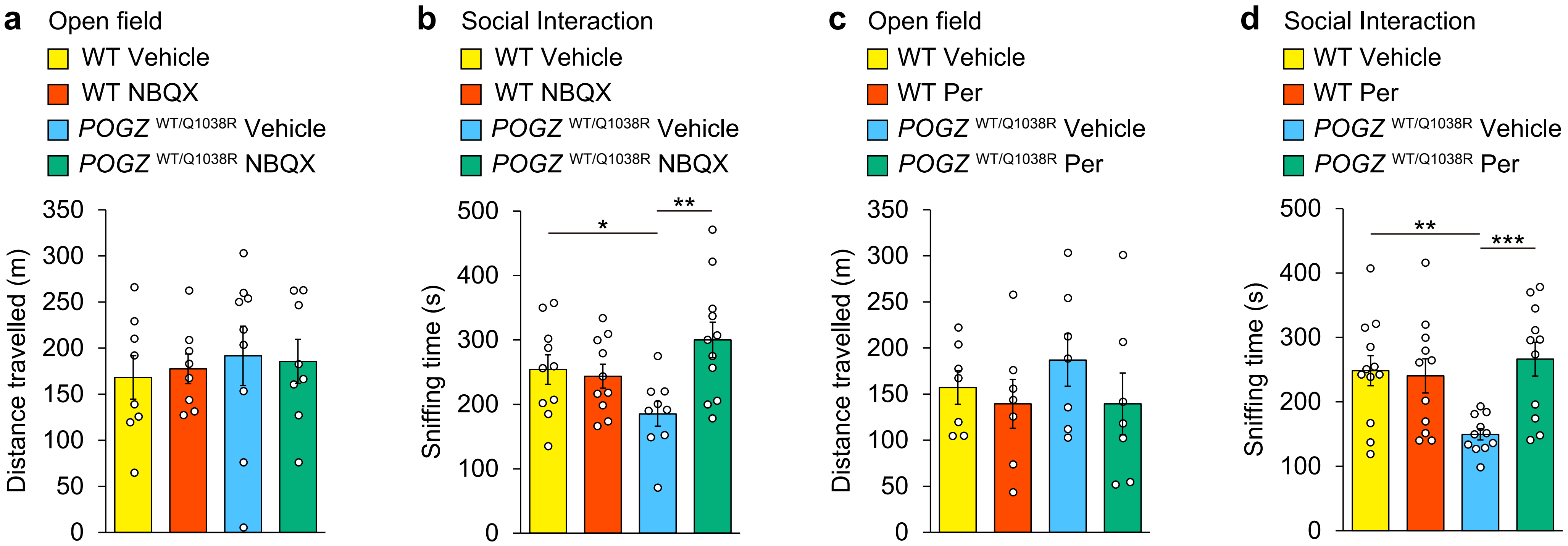

Supplementary Fig. 16 | **NBQX and perampanel treatment restore the impaired social interaction of *POGZ*$^{WT/Q1038R}$ mice. a**, Distance travelled in the open-field test 30 min after NBQX treatment (10 mg per kg) (WT Vehicle, n = 8; WT NBQX, n = 8; *POGZ*$^{WT/Q1038R}$ Vehicle, n = 9; *POGZ*$^{WT/Q1038R}$ NBQX, n = 8). **b**, Time spent sniffing in the reciprocal social interaction test 30 min after NBQX treatment (10 mg per kg) (WT Vehicle, n = 10; WT NBQX, n = 10; *POGZ*$^{WT/Q1038R}$ Vehicle, n = 9; *POGZ*$^{WT/Q1038R}$ NBQX, n = 11). **c**, Distance travelled in the open-field test 30 min after perampanel treatment (3 mg per kg) (WT Vehicle, n = 7; WT perampanel, n = 7; *POGZ*$^{WT/Q1038R}$ Vehicle, n = 7; *POGZ*$^{WT/Q1038R}$ perampanel, n = 7). **d**, Perampanel treatment (3 mg per kg) restored the impaired social interaction of *POGZ*$^{WT/Q1038R}$ mice (WT Vehicle, n = 12; WT perampanel, n = 11; *POGZ*$^{WT/Q1038R}$ Vehicle, n = 11; *POGZ*$^{WT/Q1038R}$ perampanel, n = 11). WT, wild-type; Per, perampanel. Two-way ANOVA followed by Bonferroni-Dunn *post hoc* tests; **a**, $F_{1, 29}$ = 0.09312; **b**, $F_{1, 36}$ = 7.410; **c**, $F_{1, 24}$ = 0.3024; **d**, $F_{1, 41}$ = 7.658. *$P < 0.05$, **$P < 0.01$, ***$P < 0.001$. Data are presented as the mean ± s.e.m.

Supplementary Table 1 *De novo* mutations in *POGZ*

Subject ID	Position (GRCh37)	Mutation (NM_015100.4)	Amino-acid change	Case	Reference
EE6	151402109	c.538C>T	Q180X	ASD	Stessman, H.A.F. et al., Am J Hum Genet., 2016
3	151400625	c.833C>G	S278X	ASD/ID	White J. et al., Genome Med., 2016
14483.p1	151400436	c.941G>A	S314N	ASD	Iossifov I. et al., Nature, 2014
UMCN5	151397464	c.1152dup	R385Sfs*4	ID	Stessman, H.A.F. et al., Am J Hum Genet., 2016
DDD4K.00380	151397434	c.1181_1182insAT	M394Ifs*76	Unclassified NDDs	DDD Study, Nature, 2017*
EE5	151396736	c.1212C>A	Y404X	ASD	Stessman, H.A.F. et al., Am J Hum Genet., 2016
-	151396670_151396671	c.1277_1278insC	E427X	ID	Tan B. et al., J Hum Genet., 2016
14551.p1	151384237	c.1790A>G	Y597C	ASD	Iossifov I. et al., Nature, 2014
FR5	151384217	c.1810G>T	E604X	ID	Stessman, H.A.F. et al., Am J Hum Genet., 2016
2-1402-003	151384104	c.1923C>G	H641Q	ASD	Yuen R. et al., Nat. Neurosci., 2017
UMCN7	151381211	c.2020delC	R674Vfs*9	ASD/ID	Stessman, H.A.F. et al., Am J Hum Genet., 2016
EE4	151381211	c.2020delC	R674Vfs*9	ID	Stessman, H.A.F. et al., Am J Hum Genet., 2016
1	151381022_151381025	c.2094_2097dupAACT	V700Nfs*7	Unclassified NDDs	Ye Y. et al., Cold Spring Harb Mol Case Stud., 2015
EE3	151380920_151380922	c.2197_2199delGTC	V733del	ID	Stessman, H.A.F. et al., Am J Hum Genet., 2016
UMCN4	151380688	c.2263delG	E755Sfs*36	ID	Stessman, H.A.F. et al., Am J Hum Genet., 2016
EE8	151380660	c.2291delC	P764Lfs*27	ASD	Stessman, H.A.F. et al., Am J Hum Genet., 2016
DDD4K.03076	151380641	c.2310C>G	Y770X	Unclassified NDDs	DDD Study, Nature, 2017*
1	151380630_151380627	c.2321_2324delCTCT	S774Cfs*16	ID	White J. et al., Genome Med., 2016
EE10	151379747	c.2396G>A	S799N	ASD	Stessman, H.A.F. et al., Am J Hum Genet., 2016
FR2	151379743	c.2400dupC	K801Qfs*7	ID	Stessman, H.A.F. et al., Am J Hum Genet., 2016
10C102646	151379469	c.2459_2462dupGTAC	F822Yfs*43	ASD	Neale B.M. et al., Nature, 2012
EE9Ψ	151379431	c.2501delT	L834Wfs*20	ID	Stessman, H.A.F. et al., Am J Hum Genet., 2016
7	151379418	c.2514dupC	S839Lfs*25	Unclassified NDDs	Ye Y. et al., Cold Spring Harb Mol Case Stud., 2015
FR4	151378937	c.2574delT	H858Qfs*13	ASD/ID	Stessman, H.A.F. et al., Am J Hum Genet., 2016
UMCN1	151378921	c.2590C>T	R864X	ID	Stessman, H.A.F. et al., Am J Hum Genet., 2016
DDD4K.02675	151378800	c.2711T>A	L904X	Unclassified NDDs	DDD study, Nature, 2015*
3	151378761	c.2750dupC	P918Tfs*26	Unclassified NDDs	Ye Y. et al., Cold Spring Harb Mol Case Stud., 2015
2	151378748	c.2763dupC	T922Hfs*22	ID	White J. et al., Genome Med., 2016
5	151378731	c.2780dupT	L927Pfs*17	ASD/ID	White J. et al., Genome Med., 2016
-	151378691	c.2820insG	N941Efs*3	ASD/ID	Dentici M.L. et al., Am J Med Genet A., 2017
FR3	151378675	c.2836delG	D946Mfs*12	ASD/ID	Stessman, H.A.F. et al., Am J Hum Genet., 2016
4	151378576	c.2935C > T	R979X	ID	White J. et al., Genome Med., 2016
FR6	151378510	c.3001C>T	R1001X	ASD/ID	Stessman, H.A.F. et al., Am J Hum Genet., 2016
DDD4K.00566	151378510	c.3001C>T	R1001X	Unclassified NDDs	DDD Study, Nature, 2017*
13627.p1	151378489	c.3022C>T	R1008X	ASD	Iossifov I. et al., Nature, 2014
2	151378480	c.3031C > T	Q1011X	Unclassified NDDs	Ye Y. et al., Cold Spring Harb Mol Case Stud., 2015
UMCN10	151378471	c.3040C>T	Q1014X	ID	Stessman, H.A.F. et al., Am J Hum Genet., 2016
4	151378470	c.3041delA	Q1014Rfs*5	Unclassified NDDs	Ye Y. et al., Cold Spring Harb Mol Case Stud., 2015
-	151378393	c.3118G>A	E1040K	ASD	Fukai R. et al., J Hum Genet., 2015
P1381	151378386	c.3125A>G	Q1042R	ASD	Hashimoto R. et al., J Hum Genet., 2016
EE7	151378372	c.3139G>T	E1047X	ASD	Stessman, H.A.F. et al., Am J Hum Genet., 2016
DDD4K.01196	151378156	c.3354delC	L1119Cfs*3	Unclassified NDDs	DDD study, Nature, 2015*
UMCN3	151378055_151378054	c.3456_3457delAG	E1154Tfs*4	ID	Stessman, H.A.F. et al., Am J Hum Genet., 2016
UMCN9	151378055_151378054	c.3456_3457delAG	E1154Tfs*4	ID	Stessman, H.A.F. et al., Am J Hum Genet., 2016
13398	151377904	c.3600_3607dupTGATGACG	E1203Vfs*28	ASD	DDD Study, Nature, 2017*
DDD4K.03715	151377883	c.3628A>C	T1210P	Unclassified NDDs	DDD Study, Nature, 2017*
UMCN8	151377664	c.3847C>T	Q1283X	ID	Stessman, H.A.F. et al., Am J Hum Genet., 2016
181481	151414613	c. 68A>G	D23G	Control	De Rubeis S. et al., Nature, 2014
304150	151403194	c. 407A>G	N136S	Control	De Rubeis S. et al., Nature, 2014
04C37021A	151402172	c. 475A>G	N159D	Control	De Rubeis S. et al., Nature, 2014
373385	151400355	c. 1022A>G	N341S	Control	De Rubeis S. et al., Nature, 2014
11999.s1	151397495	c.1121G>A	R374Q	Control	Sanders S. et al., Neuron, 2015
339733	151397480	c. 1136G>A	R379Q	Control	De Rubeis S. et al., Nature, 2014
DDD_MAIN5354965	151396611	c. 1377C>T	P446L	Control	De Rubeis S. et al., Nature, 2014
94124	151396443	c. 1505G>A	R502K	Control	De Rubeis S. et al., Nature, 2014
185197	151396443	c. 1505G>A	R502K	Control	De Rubeis S. et al., Nature, 2014
05C43449A	151384780	c. 2086G>A	V591I	Control	De Rubeis S. et al., Nature, 2014
252240	151384132	c. 1895A>G	N632S	Control	De Rubeis S. et al., Nature, 2014
328789	151384132	c. 1895A>G	N632S	Control	De Rubeis S. et al., Nature, 2014
347306	151384132	c. 1895A>G	N632S	Control	De Rubeis S. et al., Nature, 2014
572608	151384132	c. 1895A>G	N632S	Control	De Rubeis S. et al., Nature, 2014
514341	151381211	c. 2020C>T	R674C	Control	De Rubeis S. et al., Nature, 2014
UK10K_GS5336859	151379753	c. 2390G>A	R797Q	Control	De Rubeis S. et al., Nature, 2014
05C39335A	151379450	c. 2482G>A	D828N	Control	De Rubeis S. et al., Nature, 2014
476929	151378497	c. 3014G>A	R1005H	Control	De Rubeis S. et al., Nature, 2014
UK10K_SKUSE5080294	151378497	c. 3014G>A	R1005H	Control	De Rubeis S. et al., Nature, 2014
DDD_MAIN5318548	151378358	c. 3153C>G	F1051L	Control	De Rubeis S. et al., Nature, 2014
451927	151378260	c. 3251A>G	H1084R	Control	De Rubeis S. et al., Nature, 2014
04C27862A	151378203	c. 3308T>A	L1103H	Control	De Rubeis S. et al., Nature, 2014
200098	151377648	c. 3863C>T	A1288V	Control	De Rubeis S. et al., Nature, 2014
DDD_MAIN5247205	151377648	c. 3863C>T	A1288V	Control	De Rubeis S. et al., Nature, 2014
373775	151377301	c. 4210G>A	D1404N	Control	De Rubeis S. et al., Nature, 2014

*DDD : Deciphering Developmental Disorders

Supplementary Table 2 MISSION shRNA TRC1 vectors

shRNA	TRC number	Hairpin Sequence (5' flanking-Sense Strand-Loop-Antisense Strand-3' flanking)
shPOGZ1	TRCN0000098925	CCGG-CCTGTGAATTTGTGGGTTATT-CTCGAG-AATAACCCACAAATTCACAGG-TTTTTG
shPOGZ2	TRCN0000098926	CCGG-GCCAACAACAATGCTGGTAAT-CTCGAG-ATTACCAGCATTGTTGTTGGC-TTTTTG
shPOGZ3	TRCN0000098927	CCGG-CCATGAAGATACTCGGCATTT-CTCGAG-AAATGCCGAGTATCTTCATGG-TTTTTG
shPOGZ4	TRCN0000098929	CCGG-GCCAAACAGTTAGACCAATTA-CTCGAG-TAATTGGTCTAACTGTTTGGC-TTTTTG

Supplementary Table 3 miR30-based shRNA vector

$shRNA^{miR30}$	Hairpin Sequence (5' flanking-Sense Strand-Loop-Antisense Strand-3' flanking)
$shPOGZ^{miR30}$	TGCTGTTGACAGTGAGCG-AGGCCCGAATCTTCAGTGAAAG-TAGTGAAGCCACAGATGTA-CTTTCACTGAAGATTCGGGCCC-TGCCTACTGCCTCGGA

Supplementary Table 4 | RNA-sequencing in NSCs derived from the ASD patient carrying the Q1042R mutation of POGZ and the unaffected healthy control. Fragments per kilobase of exon per million mapped fragments (FPKM) of each transcript and fold change between the patient-derived NSCs and control NSCs. Commonly differentially expressed genes annotated to "neurogenesis (GO: 0022008)" between human and mice (78 out of 913 genes annotated to GO: 0022008) are lsited.

			Control NSC	Patient NSC	Control NSC vs Patient NSC
**Symbol**	**Description**	**EntrezGene ID**	**FPKM**	**FPKM**	**Fold Change**
CTHRC1	collagen triple helix repeat containing 1	115908	0.010	0.320	32.000
NKX6-2	NK6 homeobox 2	84504	0.060	1.440	24.000
S1PR5	sphingosine-1-phosphate receptor 5	53637	0.010	0.100	10.000
GSX2	GS homeobox 2	170825	0.420	3.030	7.214
DSCAML1	Down syndrome cell adhesion molecule like 1	57453	0.010	0.060	6.000
ISL2	insulin related protein 2 (islet 2)	64843	0.030	0.120	4.000
FGF8	fibroblast growth factor 8	2253	0.140	0.490	3.500
GABRR2	gamma-aminobutyric acid (GABA) C receptor, subunit rho 2	2570	0.050	0.160	3.200
PRKCH	protein kinase C, eta	5583	0.010	0.030	3.000
PLP1	proteolipid protein (myelin) 1	5354	17.860	44.320	2.482
SCN1B	sodium channel, voltage-gated, type I, beta	6324	0.120	0.280	2.333
CLCF1	cardiotrophin-like cytokine factor 1	23529	0.700	1.630	2.329
EGR2	early growth response 2	1959	0.370	0.840	2.270
VAX2	ventral anterior homeobox containing gene 2	25806	6.240	13.540	2.170
TLR2	toll-like receptor 2	7097	0.150	0.310	2.067
EPOR	erythropoietin receptor	2057	0.650	1.330	2.046
GSX1	GS homeobox 1	219409	10.170	20.580	2.024
TSHR	thyroid stimulating hormone receptor	7253	0.050	0.100	2.000
POSTN	periostin, osteoblast specific factor	10631	0.010	0.020	2.000
GFAP	glial fibrillary acidic protein	2670	0.010	0.020	2.000
DSCAM	Down syndrome cell adhesion molecule like 1	1826	0.010	0.020	2.000
JAG2	jagged 2	3714	0.660	1.300	1.970
NPTXR	neuronal pentraxin receptor	23467	1.960	3.810	1.944
DTX1	deltex 1 homolog (Drosophila)	1840	1.320	2.540	1.924
CHRDL1	chordin-like 1	91851	17.760	33.910	1.909
ADORA2A	adenosine A2a receptor	135	0.950	1.800	1.895
CRTAC1	cartilage acidic protein 1	55118	0.780	1.370	1.756
SLITRK3	SLIT and NTRK-like family, member 3	22865	0.170	0.290	1.706
SLC8A3	solute carrier family 8 (sodium/calcium exchanger), member 3	6547	0.780	0.460	1.696
CDH11	cadherin 11	1009	4.090	6.910	1.689
PAK3	p21 protein (Cdc42/Rac)-activated kinase 3	5063	4.580	7.680	1.677
CDHR1	cadherin-related family member 1	92211	2.530	4.190	1.656
FLRT1	fibronectin leucine rich transmembrane protein 1	23769	0.170	0.280	1.647
ISLR2	immunoglobulin superfamily containing leucine-rich repeat 2	57611	0.270	0.430	1.593
BDNF	brain-derived neurotrophic factor	627	0.090	0.140	1.556
AMIGO3	adhesion molecule with Ig like domain 3	386724	3.800	5.870	1.545
FAS	Fas (TNF receptor superfamily member 6)	355	0.790	1.180	1.494
RTN4R	reticulon 4 receptor	65078	12.600	18.810	1.493
ATXN1	ataxin 1	6310	0.240	0.350	1.458
MMP24	matrix metallopeptidase 24	10893	0.770	1.120	1.455
NPY	neuropeptide Y	4852	1.790	2.590	1.447
DCX	doublecortin	1641	31.780	42.640	1.342
MDK	midkine	4192	619.510	829.900	1.340
GPRIN1	G protein-regulated inducer of neurite outgrowth 1	114787	15.840	21.110	1.333
TEAD3	TEA domain family member 3	7005	12.650	16.840	1.331
NTNG2	netrin G2	84628	3.940	5.230	1.327
CAV1	caveolin 1, caveolae protein	857	0.370	0.490	1.324
TTBK1	tau tubulin kinase 1	84630	1.800	2.360	1.311
GPR173	G-protein coupled receptor 173	54328	12.300	15.860	1.289
RARB	retinoic acid receptor, beta	5915	7.440	9.060	1.218
CYB5D2	cytochrome b5 domain containing 2	124936	11.390	13.810	1.212
STMN2	stathmin-like 2	11075	98.920	118.870	1.202
CEP290	centrosomal protein 290	80184	6.060	4.800	-1.263
LAMA2	laminin, alpha 2	3908	0.500	0.390	-1.282
RND1	Rho family GTPase 1	27289	6.920	5.200	-1.331
CHRNB2	cholinergic receptor, nicotinic, beta polypeptide 2 (neuronal)	1141	3.480	2.590	-1.344
NEXN	nexilin	91624	2.560	1.900	-1.347
ABLIM1	actin-binding LIM protein 1	3983	10.150	7.110	-1.428
SCARF1	scavenger receptor class F, member 1	8578	0.400	0.280	-1.429
SRRM4	serine/arginine repetitive matrix 4	84530	9.270	6.430	-1.442
GHRL	ghrelin	51738	0.060	0.040	-1.500
PDGFB	platelet derived growth factor, B polypeptide	5155	0.060	0.040	-1.500
WNT7B	wingless-related MMTV integration site 7B	7477	6.120	3.740	-1.636
ERBB4	v-erb-a erythroblastic leukemia viral oncogene homolog 4 (avian)	2066	3.170	1.930	-1.642
DLX2	distal-less homeobox 2	1746	0.120	0.070	-1.714
KDR	kinase insert domain protein receptor	3791	0.120	0.070	-1.714
ITGA3	integrin alpha 3	3675	0.390	0.220	-1.773
ATP8A2	ATPase, aminophospholipid transporter-like, class I, type 8A, member 2	51761	0.670	0.350	-1.914
COBL	cordon-bleu	23242	0.150	0.070	-2.143
P2RY2	purinergic receptor P2Y, G-protein coupled 2	5029	0.150	0.070	-2.143
GRID2	glutamate receptor, ionotropic, delta 2	2895	1.430	0.660	-2.167
NEUROD1	neurogenic differentiation 1	4760	2.100	0.750	-2.800
SHH	sonic hedgehog	6469	0.030	0.010	-3.000
NCMAP	noncompact myelin associated protein	400746	0.030	0.010	-3.000
RGS6	regulator of G-protein signaling 6	9628	0.630	0.150	-4.200
MMP14	matrix metallopeptidase 14 (membrane-inserted)	4323	1.850	0.420	-4.405
LPAR3	lysophosphatidic acid receptor 3	23566	0.930	0.100	-9.300
BCL11A	B cell CLL/lymphoma 11A (zinc finger protein)	53335	0.660	0.010	-66.000

Supplementary Table 5 | RNA-sequencing in NSCs derived from the E16.5 embryonic cerebral cortex of *POGZ* $^{WT/Q1038R}$ Mice and WT Mice. FPKM of each transcript and fold change between the NSCs derived from *POGZ* $^{WT/Q1038R}$ embryos and WT embryos. Commonly differentially expressed genes annotated to "neurogenesis (GO:0022008)" between human and mice (78 out of 251 genes annotated to GO: 0022008) are lsited.

			NSC (WT mice)	NSC (*POGZ* $^{WT/Q1038R}$ mice)	NSC (WT mice) vs NSC (*POGZ* $^{WT/Q1038R}$ mice)
**Symbol**	**Description**	**EntrezGene ID**	**FPKM**	**FPKM**	**Fold Change**
Islr2	immunoglobulin superfamily containing leucine-rich repeat 2	320563	0.122	0.447	3.665
Npy	neuropeptide Y	109648	0.222	0.751	3.383
Fgf8	fibroblast growth factor 8	14179	0.094	0.237	2.373
Rtn4r	reticulon 4 receptor	65079	0.314	0.683	2.175
Jag2	jagged 2	16450	0.272	0.592	2.175
Tlr2	toll-like receptor 2	24088	0.085	0.215	2.149
Stmn2	stathmin-like 2	20257	0.397	0.783	1.973
Isl2	insulin related protein 2 (islet 2)	104360	0.136	0.269	1.973
Clcf1	cardiotrophin-like cytokine factor 1	56708	0.858	1.658	1.933
Nptxr	neuronal pentraxin receptor	73340	2.164	4.046	1.870
Mmp24	matrix metallopeptidase 24	17391	0.364	0.655	1.801
Ntng2	netrin G2	171171	1.070	1.870	1.748
Gpr173	G-protein coupled receptor 173	70771	1.666	2.902	1.742
Atxn1	ataxin 1	20238	0.168	0.290	1.728
Crtac1	cartilage acidic protein 1	72832	0.807	1.391	1.724
Fas	Fas (TNF receptor superfamily member 6)	14102	2.573	4.303	1.672
Prkch	protein kinase C, eta	18755	0.073	0.164	1.635
Nkx6-2	NK6 homeobox 2	14912	0.681	1.094	1.607
Slitrk3	SLIT and NTRK-like family, member 3	386750	1.103	1.740	1.578
Postn	periostin, osteoblast specific factor	50706	0.179	0.281	1.571
Gsx1	GS homeobox 1	14842	1.911	2.991	1.565
Scn1b	sodium channel, voltage-gated, type I, beta	20266	6.633	9.915	1.495
Cthrc1	collagen triple helix repeat containing 1	68588	0.625	0.926	1.480
Tshr	thyroid stimulating hormone receptor	22095	0.127	0.185	1.450
Ttbk1	tau tubulin kinase 1	106763	1.915	2.762	1.442
Cdh11	cadherin 11	12552	2.200	3.107	1.412
Vax2	ventral anterior homeobox containing gene 2	24113	0.219	0.308	1.410
Gabrr2	gamma-aminobutyric acid (GABA) C receptor, subunit rho 2	14409	0.139	0.196	1.410
S1pr5	sphingosine-1-phosphate receptor 5	94226	0.033	0.138	1.382
Mdk	midkine	17242	2.688	3.679	1.369
Rarb	retinoic acid receptor, beta	218772	0.961	1.306	1.358
Slc8a3	solute carrier family 8 (sodium/calcium exchanger), member 3	110893	2.257	3.030	1.343
Dcx	doublecortin	13193	0.346	0.462	1.335
Adora2a	adenosine A2a receptor	11540	0.289	0.380	1.316
Plp1	proteolipid protein (myelin) 1	18823	0.928	1.218	1.312
Cav1	caveolin 1, caveolae protein	12389	4.490	5.842	1.301
Gfap	glial fibrillary acidic protein	14580	0.000	0.129	1.291
Chrdl1	chordin-like 1	83453	12.893	16.430	1.274
Dtx1	deltex 1 homolog (Drosophila)	14357	6.862	8.723	1.271
Gsx2	GS homeobox 2	14843	0.821	1.041	1.269
Cyb5d2	cytochrome b5 domain containing 2	192986	2.965	3.761	1.269
Pak3	p21 protein (Cdc42/Rac)-activated kinase 3	18481	1.948	2.461	1.263
Amigo3	adhesion molecule with Ig like domain 3	320844	1.376	1.705	1.239
Cdhr1	cadherin-related family member 1	170677	2.317	2.870	1.239
Bdnf	brain derived neurotrophic factor	12064	0.511	0.632	1.236
Egr2	early growth response 2	13654	3.334	4.104	1.231
Dscaml1	Down syndrome cell adhesion molecule like 1	114873	0.369	0.454	1.230
Flrt1	fibronectin leucine rich transmembrane protein 1	396184	0.357	0.436	1.222
Gprin1	G protein-regulated inducer of neurite outgrowth 1	26913	6.309	7.682	1.218
Tead3	TEA domain family member 3	21678	3.678	4.475	1.217
Epor	erythropoietin receptor	13857	0.323	0.390	1.208
Dscam	Down syndrome cell adhesion molecule	13508	6.720	8.091	1.204
Ablim1	actin-binding LIM protein 1	226251	1.212	1.010	-1.200
Srrm4	serine/arginine repetitive matrix 4	68955	0.720	0.600	-1.201
Wnt7b	wingless-related MMTV integration site 7B	22422	12.637	10.472	-1.207
Cep290	centrosomal protein 290	216274	2.456	1.937	-1.268
Itga3	integrin alpha 3	16400	0.225	0.177	-1.273
Chrnb2	cholinergic receptor, nicotinic, beta polypeptide 2 (neuronal)	11444	0.534	0.415	-1.287
Bcl11a	B cell CLL/lymphoma 11A (zinc finger protein)	14025	2.028	1.575	-1.287
Dlx2	distal-less homeobox 2	13392	2.970	2.288	-1.298
Neurod1	neurogenic differentiation 1	18012	0.360	0.277	-1.301
Mmp14	matrix metallopeptidase 14 (membrane-inserted)	17387	47.957	36.645	-1.309
Nexn	nexilin	68810	10.214	7.700	-1.327
Rnd1	Rho family GTPase 1	223881	2.547	1.901	-1.340
Pdgfb	platelet derived growth factor, B polypeptide	18591	1.449	1.064	-1.362
Shh	sonic hedgehog	20423	0.213	0.154	-1.379
Scarf1	scavenger receptor class F, member 1	380713	0.140	0.024	-1.396
Kdr	kinase insert domain protein receptor	16542	0.371	0.265	-1.397
Grid2	glutamate receptor, ionotropic, delta 2	14804	0.159	0.112	-1.419
Cobl	cordon-bleu	12808	0.152	0.105	-1.445
P2ry2	purinergic receptor P2Y, G-protein coupled 2	18442	0.428	0.294	-1.455
Lama2	laminin, alpha 2	16773	0.543	0.372	-1.457
Erbb4	v-erb-a erythroblastic leukemia viral oncogene homolog 4 (avian)	13869	0.849	0.562	-1.511
Ncmap	noncompact myelin associated protein	230822	0.241	0.153	-1.576
Lpar3	lysophosphatidic acid receptor 3	65086	0.163	0.028	-1.635
Atp8a2	ATPase, aminophospholipid transporter-like, class I, type 8A, member 2	50769	0.172	0.091	-1.720
Rgs6	regulator of G-protein signaling 6	50779	0.216	0.078	-2.164
Ghrl	ghrelin	58991	0.703	0.198	-3.547
