# Supplementary material for: Pathogenic POGZ mutation causes impaired cortical development and reversible autism-like phenotypes
Source: Nat Commun. 2020 Feb 26;11:859. doi: 10.1038/s41467-020-14697-z (PMC7044294; doi:10.1038/s41467-020-14697-z)
Supplement: Supplementary file 3 — Reporting Summary [file 41467_2020_14697_MOESM3_ESM.pdf]

nature research

Corresponding author(s): Takanobu Nakazawa

Last updated by author(s): Jan 4, 2020

Reporting Summary

Nature Research wishes to improve the reproducibility of the work that we publish. This form provides structure for consistency and transparency in reporting. For further information on Nature Research policies, see Authors & Referees and the Editorial Policy Checklist .

Statistics

For all statistical analyses, confirm that the following items are present in the figure legend, table legend, main text, or Methods section.

n/a	Confirmed	
☐	☒	The exact sample size (n) for each experimental group/condition, given as a discrete number and unit of measurement
☐	☒	A statement on whether measurements were taken from distinct samples or whether the same sample was measured repeatedly
☐	☒	The statistical test(s) used AND whether they are one- or two-sided<br>*Only common tests should be described solely by name; describe more complex techniques in the Methods section.*
☒	☐	A description of all covariates tested
☐	☒	A description of any assumptions or corrections, such as tests of normality and adjustment for multiple comparisons
☐	☒	A full description of the statistical parameters including central tendency (e.g. means) or other basic estimates (e.g. regression coefficient) AND variation (e.g. standard deviation) or associated estimates of uncertainty (e.g. confidence intervals)
☐	☒	For null hypothesis testing, the test statistic (e.g. F, t, r) with confidence intervals, effect sizes, degrees of freedom and P value noted<br>*Give P values as exact values whenever suitable.*
☒	☐	For Bayesian analysis, information on the choice of priors and Markov chain Monte Carlo settings
☒	☐	For hierarchical and complex designs, identification of the appropriate level for tests and full reporting of outcomes
☒	☐	Estimates of effect sizes (e.g. Cohen's d, Pearson's r), indicating how they were calculated

Our web collection on statistics for biologists contains articles on many of the points above.

Software and code

Policy information about availability of computer code

Data collection	LAS4000 image analyzer (biochemistry), CFX96 real-time PCR detection system (biochemistry), FV1000, BZ-9000 and ToxInsight microscopes (histology), iMARK Microplate Reader (biochemistry), Avisoft-SASLab Pro software (behavior), Anymaze software (behavior), Igor Pro 7 (electrophysiology), Multiclamp 700B amplifier (electrophysiology) and ScanXmate-E090S (CT analysis) were used for data collection.
Data analysis	Stat-View (version 5.0), R (version 3.4.1), NIH ImageJ (version 1.5b), OsiriX, TRI/3D-BON and Photoshop CS6 was used to analyze the data.

For manuscripts utilizing custom algorithms or software that are central to the research but not yet described in published literature, software must be made available to editors/reviewers. We strongly encourage code deposition in a community repository (e.g. GitHub). See the Nature Research guidelines for submitting code & software for further information.

Data

Policy information about availability of data

All manuscripts must include a data availability statement. This statement should provide the following information, where applicable:

- Accession codes, unique identifiers, or web links for publicly available datasets
- A list of figures that have associated raw data
- A description of any restrictions on data availability

The datasets generated during the current study are available from the corresponding authors on reasonable request.

Field-specific reporting

Please select the one below that is the best fit for your research. If you are not sure, read the appropriate sections before making your selection.

Life sciences study design

All studies must disclose on these points even when the disclosure is negative.

Sample size	We did not use statistical methods to predetermine the sample sizes, but the sample sizes in this study are similar to those generally employed in this field.
Data exclusions	All obtained data were included in the analysis.
Replication	All studies have been repeated with similar results. We included detailed methods in the manuscript.
Randomization	No randomization was employed in this study.
Blinding	All histological, cell biological, behavioral and electrophysiological experiments were blindly performed.

Reporting for specific materials, systems and methods

We require information from authors about some types of materials, experimental systems and methods used in many studies. Here, indicate whether each material, system or method listed is relevant to your study. If you are not sure if a list item applies to your research, read the appropriate section before selecting a response.

Materials & experimental systems

n/a	Involved in the study	
☐	☒	Antibodies
☐	☒	Eukaryotic cell lines
☒	☐	Palaeontology
☐	☒	Animals and other organisms
☐	☒	Human research participants
☒	☐	Clinical data

Methods

n/a	Involved in the study	
☒	☐	ChIP-seq
☒	☐	Flow cytometry
☒	☐	MRI-based neuroimaging

Antibodies

Antibodies used

rabbit anti-POGZ (SIGMA-Aldrich, #AV39172, 1:1000)
mouse anti-Myc (9E10) (Santa Cruz Biotechnology, CA, USA, #sc-40, 1:400)
rabbit anti-Lamin A/C (Cell Signaling Technology, MD, USA, #2032, 1:1000)
mouse anti- -Tubulin (DM1A) (SIGMA-Aldrich, #T9026, 1:5000)
rabbit anti-GFP (MBL, Aichi, Japan, #598, 1:200)
chicken anti-GFP (Abcam, Cambridge, UK, #ab13970, 1:500)
rabbit anti-PAX6 (BioLegend, CA, USA, #901301, 1:50)
rat anti-SOX2 (Molecular Probe, OA, USA, #A-24339, 1:50)
rabbit anti-TBR2 (Abcam, #ab23345, 1:50)
mouse anti-SATB2 (Abcam, #ab51502, 1:50)
rabbit anti-CUX1 (Santa Cruz Biotechnology, #sc-13024, 1:50)
rat anti-CTIP2 (Abcam, #ab18465, 1:50)
rabbit anti-TBR1 (Proteintech, IL, USA, #20932-1-AP, 1:50)
mouse anti-BrdU (Abcam, #ab6326, 1:40)
rabbit anti-MAP2 (Merck Millipore, MA, USA, #AB5622, 1:200)
mouse anti-NESTIN (Merck Millipore, #MAB5326, 1:1000)
HRP-conjugated goat anti-rabbit IgG (Santa Cruz Biotechnology, #sc-2004, 1:1000)
HRP-conjugated goat anti-mouse IgG (Santa Cruz Biotechnology, #sc-2005, 1:1000)
AP-conjugated goat anti-rabbit IgG (Santa Cruz Biotechnology, #sc-2007, 1:1000)
AP-conjugated goat anti-mouse IgG (Santa Cruz Biotechnology, #sc-2008, 1:1000)
biotinylated goat anti-rabbit IgG (Vector Labs, CA, USA, #BA-1000, 1:200)
biotinylated goat anti-mouse IgG (Vector Labs, #BA-9200, 1:200)
Alexa Fluor 488-conjugated goat anti-rabbit IgG (Life Technologies, CA, USA, #A-11008, 1:200)
Alexa Fluor 488-conjugated goat anti-chicken IgY (Life Technologies, #A-11039, 1:500)
Alexa Fluor 647-conjugated goat anti-rat IgG (Life Technologies, #A-21247, 1:200)
Alexa Fluor 594-conjugated donkey anti-mouse IgG (Jackson ImmunoResearch, PA, USA, #715-585-150, 1:250)
Stem Light Pluripotency Antibody Kit (Cell Signaling Technology, #9656S).
POD-conjugated sheep anti-Digoxigenin (DIG; Fab fragments; Roche Life Sciences, Basel, Switzerland, #11207733910, 1:250).

Validation	The antibodies were purchased by qualified vendors that provided a validation on the manufacturer's website.

Eukaryotic cell lines

Policy information about cell lines

Cell line source(s)	Mouse neuroblastoma Neuro2a cells<br>SH-SY5Y cells<br>Human iPS cell lines
Authentication	Neuro2a cells and SH-SY5Y cells: Stock of cell lines were generated after purchase. For experiments, cell lines were not kept for more than 1 consecutive month in culture.<br>iPS cell lines: Generation of iPSC lines using immortalized B cells obtained from the ASD patient carrying Q1042R POGZ mutation and from her unaffected father was performed as described previously (Nakazawa et al., Schizophr Res., 2017). Colonies of cells similar to human embryonic stem (ES) cells were clonally isolated, morphologically selected, subjected to PCR-based analysis of episomal vector loss and evaluated for expression of pluripotent markers using immunohistochemistry (Oct-4A, Sox2, TRA-1-60 and TRA-1-81).
Mycoplasma contamination	Cells were tested for mycoplasma contamination by PCR. If cells resulted positive for contamination, they were treated with mycoplasma removal agents.
Commonly misidentified lines (See ICLAC register)	No commonly misidentified cell lines have been used in this study.

Animals and other organisms

Policy information about studies involving animals; ARRIVE guidelines recommended for reporting animal research

Laboratory animals	All behavioral tests were carried out on male C57BL/6NJcl mice at 1.5-4 months of age, except the ultrasonic vocalization test (postnatal day 4) and the juvenile play test (3 weeks of age). For in utero electroporation, E14.5 embryos (ICR mice) were used. For immunohistochemistry, E16.5 and E18.5 embryos and ten-week-old male mice (C57BL/6NJcl mice) were used. For electrophysiology, ten- to eleven-week-old male mice (C57BL/6NJcl mice) were used.
Wild animals	The study did not involve wild animals.
Field-collected samples	The study did not involve samples collected from the field.
Ethics oversight	The animal experiments were performed in accordance with the guidelines for animal use issued by the Committee of Animal Experiments, Osaka University, Jikei University School of Medicine and RIKEN Tsukuba Branch, and were approved by the Committee.

Note that full information on the approval of the study protocol must also be provided in the manuscript.

Human research participants

Policy information about studies involving human research participants

Population characteristics	The patient had been diagnosed by at least two trained child psychiatrists according to the criteria in the Diagnostic and Statistical Manual of Mental Disorders, fourth edition, text revision (DSM-IV-TR) based on interviews with the patient and unstructured or semi-structured observations of her behavior. During the interview, the Pervasive Developmental Disorders Autism Society Japan Rating Scale (PARS) and the Japanese version of the Asperger's Questionnaire (AQ) were used to assist in the evaluation of ASD-specific behaviors and symptoms. The PARS is a semi-structured interview that is composed of questions in eight domains corresponding to the characteristics of children with pervasive developmental disorders (PDDs), which was developed by the Autism Society Japan. The clinicians who diagnosed the subject were trained in the use of the PARS.
Recruitment	A patient with ASD and the parents were recruited from outpatient services at Osaka University Hospital.
Ethics oversight	This study was carried out in accordance with the World Medical Association's Declaration of Helsinki and was approved by the Research Ethics Committee in Osaka University.

Note that full information on the approval of the study protocol must also be provided in the manuscript.
